# Cancer Stem Cells, not Bulk Tumor Cells, Determine Mechanisms of Resistance to SMO Inhibitors

**DOI:** 10.1158/2767-9764.CRC-22-0124

**Published:** 2022-06-06

**Authors:** Joshy George, Yaohui Chen, Nourhan Abdelfattah, Keiko Yamamoto, Thomas D. Gallup, Scott I. Adamson, Brad Rybinski, Anuj Srivastava, Parveen Kumar, Min Gyu Lee, David S. Baskin, Wen Jiang, Jong Min Choi, William Flavahan, Jeffrey H. Chuang, Betty Y.S. Kim, Jiaqiong Xu, Sung Yun Jung, Kyuson Yun

**Affiliations:** 1 The Jackson Laboratory for Genomic Medicine, Farmington, Connecticut.; 2 Department of Neurosurgery, Houston Methodist Neurological Institute and Institute for Academic Medicine, Houston, Texas.; 3 The Kenneth R. Peak Brain and Pituitary Tumor Treatment Center, Houston Methodist, Houston Texas.; 4 Department of Neurology, Houston Methodist Hospital and Houston Methodist Research Institute, Houston, Texas.; 5 The Jackson Laboratory-Mammalian Genetics, Bar Harbor, Maine.; 6 Department of Neurosurgery, The University of Texas MD Anderson Cancer Center, Houston, Texas.; 7 Department of Genetics and Genome Sciences, UConn Health, Farmington, Connecticut.; 8 Department of Internal Medicine, University of Maryland Medical Center, Baltimore, Maryland.; 9 Department of Molecular and Cellular Oncology, The University of Texas MD Anderson Cancer Center, Houston, Texas.; 10 Department of Neurosurgery, Weill Cornell Medical College, New York, New York.; 11 Department of Radiation Oncology, The University of Texas Southwestern Medical Center, Dallas, Texas.; 12 Advanced Technology Core, Mass Spectrometry Proteomics Core, Baylor College of Medicine, Houston, Texas.; 13 Department of Molecular, Cell and Cancer Biology, University of Massachusetts Chan Medical School, Worcester, Massachusetts.; 14 Center for Outcomes Research, Houston Methodist Research Institute, Houston Texas.; 15 Department of Biochemistry and Molecular Biology, Baylor College of Medicine, Houston, Texas.; 16 Department of Neurology, Weill-Cornell Medical College, New York, New York.

## Abstract

The emergence of treatment resistance significantly reduces the clinical utility of many effective targeted therapies. Although both genetic and epigenetic mechanisms of drug resistance have been reported, whether these mechanisms are stochastically selected in individual tumors or governed by a predictable underlying principle is unknown. Here, we report that the dependence of cancer stem cells (CSC), not bulk tumor cells, on the targeted pathway determines the molecular mechanism of resistance in individual tumors. Using both spontaneous and transplantable mouse models of sonic hedgehog (SHH) medulloblastoma treated with a SHH/Smoothened inhibitor (SMOi), sonidegib/LDE225, we show that genetic-based resistance occurs only in tumors that contain SHH-dependent CSCs. In contrast, SHH medulloblastomas containing SHH-dependent bulk tumor cells but SHH-independent CSCs (SI-CSC) acquire resistance through epigenetic reprogramming. Mechanistically, elevated proteasome activity in SMOi-resistant SI-CSC medulloblastomas alters the tumor cell maturation trajectory through enhanced degradation of specific epigenetic regulators, including histone acetylation machinery components, resulting in global reductions in H3K9Ac, H3K14Ac, H3K56Ac, H4K5Ac, and H4K8Ac marks and gene expression changes. These results provide new insights into how selective pressure on distinct tumor cell populations contributes to different mechanisms of resistance to targeted therapies. This insight provides a new conceptual framework to understand responses and resistance to SMOis and other targeted therapies.

**Significance:**

The mechanism by which individual tumors become resistant to targeted therapies is thought to be unpredictable. This study provides novel insights into how selective pressure on cancer stem versus bulk tumor cells drives distinct and predictable mechanisms of resistance to targeted therapies. This finding paves a way for future treatment strategies that incorporate anticipated resistance mechanisms in devising second-line therapies in a personalized manner.

## Introduction

The sonic hedgehog (SHH) pathway is a highly conserved developmental signaling pathway that is activated in many human cancers, including basal cell carcinoma (BCC), acute myeloid leukemia (AML), and medulloblastoma (1, 2). Currently, there are two FDA-approved SHH/Smoothened (SMO) inhibitors (SMOi: vismodegib and sonidegib) for treating BCCs and other cancer types in which SHH pathway activity is elevated. Unfortunately, as observed for other highly effective targeted therapies (3), a significant portion of patients with BCC initially responsive to SMOis develop resistance over time (4–6) or experience tumor rebound upon cessation of treatment (7). In clinical trials for SHH-subtype medulloblastomas, an objective response rate of 55% has been reported for sonidegib/LDE225, while vismodegib/GDC-0449 has shown an objective response rate of 17% (8), indicating that not all SHH-subtype medulloblastomas respond to SMOi treatment. A subset of these nonresponders was shown to harbor downstream mutations in the SHH pathway (9), while others developed acquired resistance over time or were inherently nonresponsive (10–16). In patients with BCC, most—albeit not all—resistant tumors harbor treatment-induced mutations in SHH pathway components (4, 5). However, only approximately 50% of SMOi-resistant medulloblastomas were shown to acquire mutations in the SHH pathway in a preclinical study (10), suggesting that additional mechanisms of resistance to SMOis must exist.

Medulloblastoma is a classical developmental cancer (2, 17) and is commonly believed to originate from cerebellar granule progenitors (CGP) that normally depend on SHH signaling for proliferation. More recent studies, however, have shown that SHH medulloblastomas can arise from either transformed neural stem cells (NSC) in the neuroepithelium or CGPs in the external granule layer (EGL) when the SHH pathway is aberrantly activated (18, 19). In fact, bulk tumor cells arising from transformed NSCs or CGPs with constitutive *Smoothened* (Smo-M2) expression are indistinguishable. However, cancer stem cells (CSC) from different cells of origin have distinct cellular characteristics, including their dependence on SHH signaling (18). CSCs retain the epigenetic memory of their cells of origin, including growth factors that they rely on for proliferation and survival. Hence, CSCs that arise from NSCs depend on bFGF and EGF instead of SHH, while CSCs that arise from transformed EGL progenitors depend on SHH signaling (18, 20, 21). Previously, others have shown that CSCs are more resistant to cytotoxic chemotherapies and radiotherapies (22, 23) and seed tumor recurrence (24, 25). However, the role of the differential mitogenic/survival pathway dependencies between bulk tumor cells and CSCs as a potential mechanism of resistance to targeted therapy has not been explored.

**FIGURE 1:**
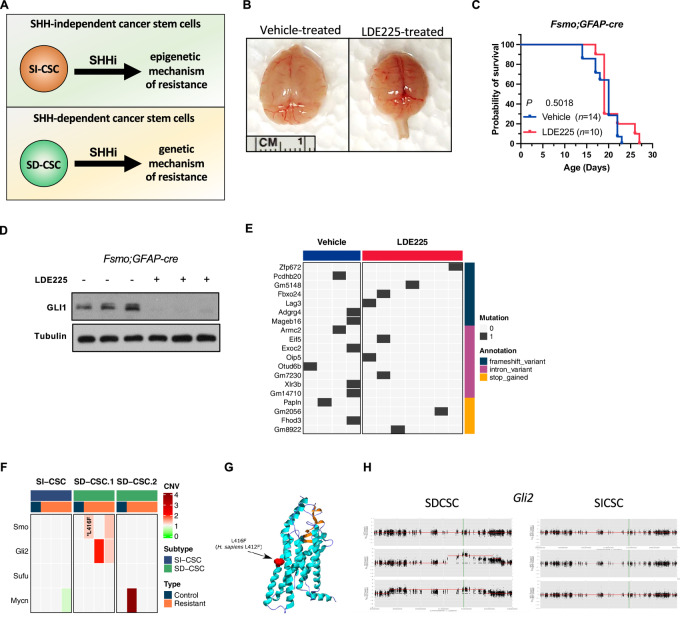
SMOi-induced mutations to SHH pathway genes only occur in medulloblastomas in which CSCs depend on the SHH signaling. **A,** A schematic of the major hypothesis tested in the study. **B,** Gross images of vehicle- and LDE225-treated *fSmoM2;hGFAP-cre* brains at harvest. **C,** Kaplan–Meier survival curve analysis showing no significant survival benefit of LDE225 treatment. **D,** GLI1 protein level is reduced in LDE-treated *fSmoM2;GFAPcre* tumors *in vivo*. **E,** A summary of high impact mutations in vehicle- and LDE225-treated *fSmoM2;hGFAP-cre* medulloblastomas. **F,** A summary of identified mutations and copy-number alterations in SHH pathway genes in SMOi-resistant SI-CSC and SD-CSC *Ptch;p53* medulloblastoma. **G,** LDE225-induced *SMO* mutation in *Ptch;p53* SD-CSC tumor. **H,** LDE225-induced *Gli2* amplification observed in two SD-CSC tumors from the same cohort, which is not observed in any SI-CSC medulloblastomas. Copy-number variation compared with vehicle-treated tumor from the same cohort.

Here, we tested our hypothesis that treatment-induced mutations in the targeted pathway (SHH) occur only in tumors in which CSCs depend on the targeted pathway. We hypothesized that in tumors where CSCs and bulk tumor cells depend on different mitogenic pathways, targeted therapy selected on the basis of bulk tumor analysis results in only a transient response (debulking) followed by resistant tumor growth due to the presence of inherently resistant CSCs. Specifically, we tested whether SMOi-resistant tumors containing SHH-dependent CSCs (SD-CSC) acquire new mutations in the SHH pathway, while SHH medulloblastomas containing SHH-independent CSCs (SI-CSC), that is, SI-CSC medulloblastomas, do not (Fig. 1A). The results of this study reveal a novel mechanism of inherent therapeutic resistance and provide a new explanation for the clinical failure of targeted therapies and the emergence of resistant tumors that do not acquire new mutations in the targeted pathway. These findings have significant clinical implications for the selection of targeted therapies, particularly for second-line therapy.

## Materials and Methods

### Mouse Models and *In Vivo* Drug Treatment

*Ptch* (*Ptch1^tm1Mps^*/J IMSR, catalog no. JAX:003081, RRID:IMSR_JAX:003081), *FSmoM2* [Gt (ROSA)26Sor<tm1 (Smo/EYFP)Amc>/J, IMSR, catalog no. JAX:005130, RRID:IMSR_JAX:005130], h*GFAP-cre* [FVB-Tg(GFAP-cre)25Mes/J, IMSR, catalog no. JAX:004600, RRID:IMSR_JAX:004600], *p53* (B6.129S2-*Trp53^tm1TyjI^*/J, IMSR catalog no. JAX:002101, RRID:IMSR_JAX:002101), NSG (NOD. Cg-*Prkdc^scid^*) *Il2rg^tm1Wjl^*/SzJ (IMSR, catalog no. JAX:005557, RRID:IMSR_JAX:005557), and B6 (C57BL/6J, IMSR catalog no. JAX:000664, RRID:IMSR_JAX:000664) mice were obtained from the JAX repository. Medulloblastoma subtype determination was performed as described previously (23). To eliminate potential spatial heterogeneity within the parental tumor, each *Ptch;p53* spontaneous medulloblastoma was first minced into a slurry, and equivalent pools of tumor cells were injected into male or female 6 to 8 weeks old NSG mice. When the tumor volume reached 100–200 mm^3^, mice were treated with 40 mg/kg LDE225 or vehicle control (oral gavage, every 3 days) until harvest. *FSmoM2;hGFAP-cre* pups were treated with LDE225 using the same dose and schedule as *Ptch;p53* MBs, starting at p2 or p3. Mice were randomly assigned to each group and were age and sex matched at the time of the experiment. Mice were housed and handled in accordance with the protocols and procedures approved by The Jackson Laboratory and the HMRI Institutional Animal Care and Usage Committees.

### Tumor Subtype Characterization

The SD-CSC and SI-CSC tumor subtype designation methods were reported previously (18). Briefly, freshly dissociated single cells were isolated from spontaneous medulloblastomas and plated at a low density (3,000 cells in 3 mL) in either TSC [modified DME/F-12 supplemented with B27 (Invitrogen) and penicillin/streptomycin] or NSC (TSC plus 20 ng/mL EGF and 10 ng/mL bFGF) medium in triplicates in 6-well plates, and secondary sphere formation was scored 5–7 days later. Tumors that formed secondary spheres only in NSC but not TSC medium were designated growth factor–dependent/SI-CSC subtype. Those that formed spheres in either NSC or TSC medium were designated as growth factor–independent/SD-CSC subtype. Those that did not form secondary spheres in either TSC or NSC medium were designated as no growth/SD-CSC subtype based on additional analyses (18).

### Primary Cells and *In Vitro* Drug Treatment

Primary tumorsphere cells were isolated from *Ptch;p53* or *FsmoM2;hGFAP-Cre* mouse medulloblastomas and cultured in serum-free, NSC medium with or without exogenous growth factors, bFGF and EGF. For SD-CSC tumor cells, we used NSC medium. SI-CSC tumorspheres were cultured in the TSC medium. Cells were treated with the indicated concentrations of inhibitors: LDE225 (10 μmol/L, catalog no. 500511 from Chemie Tek), GANT61 (10 μmol/L, catalog no. 3191 from TOCRIS Bioscience), and trichostatin A (0.01 or 0.1 μmol/L, catalog no. T8552 from SIGMA). Histone acetyltransferase inhibitor IV (10 μmol/L, catalog no. 382111 from EMD Millipore Corp.), histone acetyltransferase inhibitor VIII (10 μmol/L, catalog no. 382111 from Calbiochem), MG132 (25 μmol/L, catalog no. M7449 from Sigma), and JQ1 (0.01 or 0.1 μmol/L, provided by J. Bradner).

No established cell lines were used in this study. Cells used in this study were freshly dissociated spontaneous mouse tumors that developed in our animal facility. Mice that develop spontaneous tumors are genotyped in our lab using PCR assays before tumor harvests. In addition, our lab routinely tests for potential *Mycoplasma* infection by a PCR assay to ensure that all cultured cells are *Mycoplasma* free. Cryo-preserved primary cells used in the study were tested negative for *Mycoplasma* upon recovery.

### Whole-Exome Sequencing Analysis

Raw fastq reads from bulk exome sequencing data were mapped to the mouse reference genome GRCm38 using BWA, RRID:SCR_010910 (version 0.7.12; ref. 26), followed by removal of duplicate reads using Picard, RRID:SCR_006525 (version 2.1; http://broadinstitute.github.io/picard/). The resulting BAM files were further realigned around indels and recalibrated for base quality using GATK, RRID:SCR_001876 (version 3.5.0; ref. 27). For GATK, the known variant sites were downloaded from the Wellcome Trust Sanger Institute, v5 release REL-1505-SNPs_Indels (ftp://ftp-mouse.sanger.ac.uk/REL-1505-SNPs_Indels). Next, the recalibrated BAM files were used as tumor-normal pairs to detect somatic mutations using MuTect, RRID:SCR_000559. Finally, SnpEff, RRID:SCR_005191 (28) was used to annotate the somatic mutations, and only the variants annotated as high impact and showing no mutant reads in the normal sample were used for downstream analysis. The copy-number analysis for bulk exome datasets was performed using the Sequenza algorithm with default parameters. Only the copy-number segments greater than 1 megabase in size were used for downstream analysis.

### RNA Sequencing Analysis

RNA sequencing (RNA-seq) analysis was carried out using the in-house pipeline at The Jackson Laboratory. Trimmomatic, RRID:SCR_011848 (version 0.33) was used to remove adapters and leading and trailing low-quality bases. Reads fewer than 36 bases long were discarded. Reads with more than 50% low-quality bases overall were filtered out, and the remaining high-quality reads were then used for expression estimation. Alignment estimation of gene expression levels using the EM algorithm for paired end read data was performed using RSEM, RRID:SCR_013027 (package version 1.2.12). RSEM uses Bowtie 2 and RRID:SCR_016368 as aligners to align the mapped reads against the mm10 reference genome. Data quality control was performed using Picard, RRID:SCR_006525 (version 1.95; http://broadinstitute.github.io/picard/) and Bamtools, RRID:SCR_015987 to obtain general alignment statistics from the bam file. Analyses of aligned reads were performed using the R package edgeR, RRID:SCR_012802. Gene set enrichment pathway analysis was performed using edgeR preranked gene lists with the fgsea (fgsea, RRID:SCR_020938) and GAGE (RRID:SCR_017067) R packages or log_2_-normalized CPM (counts per million) values with the gene set variation analysis (GSVA) R package. Plots were generated using ggplot2 and RRID:SCR_014601, and heatmaps were generated using ComplexHeatmap and RRID:SCR_017270.

### Proteomics Analysis

Label-free proteome profiling was carried out as described previously (29). Briefly, the cell pellets were dissolved in 50 mmol/L ammonium bicarbonate and 1 mmol/L CaCl_2_ buffer followed by three rounds of liquid nitrogen freezing and 95°C boiling for 2 minutes. The lysate was digested by trypsin (Gendepot T9600) at 37°C overnight. The tryptic peptides were desalted prefractionated into five fractions using C18 beads. LC/MS-MS analysis was carried out using a nanoLC1200 system coupled to an Orbitrap Fusion Tribrid mass spectrometer (Thermo Fisher Scientific). One microgram of peptide was loaded onto a C18 (1.9 μm, Reprosil-Pur Basic Dr. Maisch GmbH) trap column and switched in line with a 50 mm × 150 μm analytic column packed with the same C18 beads. The peptides were eluted using a 75-minute linear gradient of 4% to 26% acetonitrile, ionized, and measured in data-dependent mode acquiring fragmentation spectra of the top 30 strongest ions and under the direct control of Xcalibur software ver. 4.0 (Thermo Fisher Scientific Xcalibur, RRID:SCR_014593). The MASCOT search engine (Mascot, RRID:SCR_014322) was used to match the spectrum to the corresponding peptide sequence searched against NCBI's mouse RefSeq protein. The Mascot results were validated with the Percolator-based *q* value in the Proteome Discoverer software [Thermo Fisher Scientific, PD2.1- (RRID:SCR_014477)]. Dynamic modification was allowed for oxidation (methionine), protein N-terminal acetylation, and deamidation (asparagine and glutamine). The maximum tolerance for precursor ions was set to 20 ppm; the fragment mass tolerance was set to 0.5 daltons; and a maximum of two missed cleavages was allowed. The calculated AUC of peptides was used to calculate iBAQ and iFOT for protein abundance based on a previous publication (29).

### Bisulfite Sequencing Analysis

Raw bisulfite sequencing reads were aligned using bismark (v0.23.1; RRID:SCR_005604; ref. 30) and bowtie2 (v2.4.4; ref. 31; RRID:SCR_016368) onto the reference mouse genome Grcm38. Subsequently, the duplicate reads that aligned to the same position and in the same orientation were removed using “deduplicate_bismark” from the bismark bisulfite mapper. Methyl-seq data were further analyzed by fusing the methylKit R package from Bioconductor (32). Principal component analysis was performed to generate a low-dimensional representation of methylation profiles using the method available from the same package.

### Gene Set Enrichment Pathway Analysis

Gene set enrichment analysis (GSEA) was performed using msmsTests::msms.edgeR() preranked gene lists with fgsea (fgsea, RRID:SCR_020938), GAGE (RRID:SCR_017067) R packages or log_2_ normalized iFOT values with GSVA R package. Plots were generated using ggplot2 (RRID:SCR_014601), and heatmaps were generated using ComplexHeatmap (RRID:SCR_017270).

### Proteasome Reporter Assay

Cell-based Proteasome-Glo Assay for Chymotrypsin-like protease activities (Promega) was performed following the manufacturer's instructions. Equal numbers of cells were plated, and the reporter assay levels were normalized to viability in each sample. All assays were repeated at least three times in triplicate, and error bars represent the SEM.

### Survival Analysis of the External Human Medulloblastoma Dataset

To assess the correlation between BRD2 or BRD4 expression and survival in the publicly available human medulloblastoma dataset (Cavalli dataset: GSE85217; ref. 33), Kaplan–Meier survival analysis was performed by stratifying patients based on median expression using the ‘‘survival’’ R package (https://github.com/therneau/survival) and plotted using the ‘‘survminer’’ R package (https://github.com/kassambara/survminer).

### Immunoblot Analysis

**FIGURE 2:**
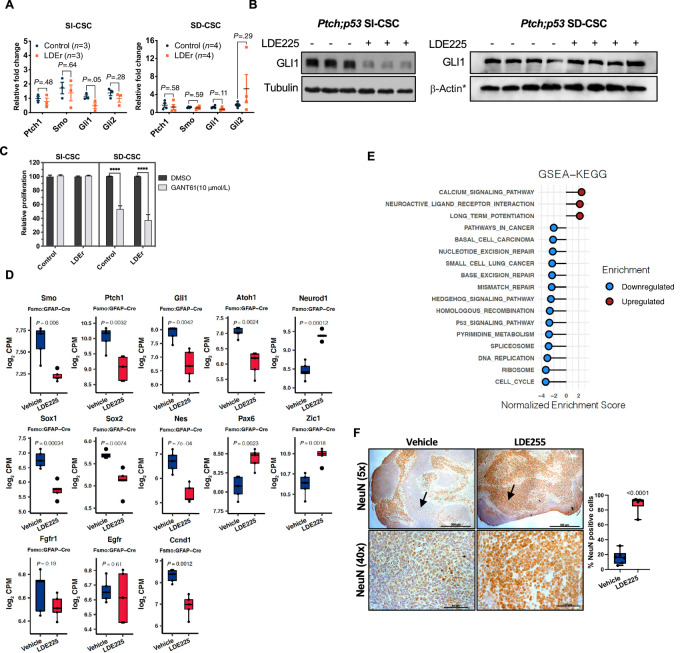
SMOi-resistant SI-CSC medulloblastomas grow independently of the SHH pathway. **A,** RT-PCR analyses of SHH pathway genes in *Ptch;p53* SI-CSC and SD-CSC medulloblastomas treated with vehicle or LDE225. Error bars represent SEM. *P* values were calculated using two-tailed Student *t* test. **B,** GLI1 protein levels in control and LDE225-resistant *Ptch;p53* SI-CSC and SD-CSC tumors. *ß-ACTIN loading control is the same as shown in Figs. 4C and F as the same blot was stripped and probed with multiple antibodies due to limited sample amounts. **C,** GANT61 (GLI inhibitor) treatment of *Ptch;p53* SI-CSC and SD-CSC tumorsphere cells *in vitro*. *N* = 3; ^****^, *P* < 0.0001, by two-tailed Student *t* test. Error bars represent SEM. **D,** RNA-seq expression levels of *SHH,* neuronal stem cell and differentiation markers in vehicle versus LDE225-treated *fSmoM2;GFAP-cre SI-CSC* medulloblastomas. Box represents log_2_-scaled CPM range in RNA-seq data. Central line represents the mean. *P* values were calculated using two-tailed Student *t* test. **E,** GSEAs (2<NES >2) of *fSmoM2;hGFAP-cre* SI-CSC tumors showing negative enrichment of the hedgehog signaling pathway, cell cycle, DNA-repair, and other gene sets and positive enrichment for gene sets associated with neuronal differentiation and function in LDE225-resistant SI-CSC tumors. **F,** Representative IHC images showing increased NEUN-expressing cells in LDE225-treated *fSmoM2;hGFAPcre* medulloblastomas. Low magnification shows overall increase in NEUN positive cells throughout the LDE-treated cerebellum. High magnification images taken from high cellular density areas. Six representative fields from three matched pairs of vehicle- versus LDE-treated samples were counted manually. Scale bar (5× = 500 μM, 40× = 50 μM). Arrows point to equivalent tumor areas with high nuclear density.

Total proteins from tissue or cells were obtained using RIPA buffer supplemented with protease and phosphatase inhibitor cocktails. A total of 30–50 μg of lysates was resolved on 10% or 15% SDS-PAGE gels, and proteins were transferred to polyvinylidene difluoride membranes (Bio-Rad, catalog no. 162-0177). Subsequent Western analyses were performed using standard procedures. Antibodies against CBP (RRID: AB_2616020, 1:1,000), GCN5 (RRID: AB_2128281, 1:3,000), PCAF (RRID: AB_2128409, 1:3,000), HDAC1 (RRID: AB_10612242, 1:2,000), HDAC2 (RRID: AB_10624871, 1:3,000), HDAC3 (RRID: AB_2118371, 1:3,000), histone H3 (RRID: AB_10544537, 1:10,000), H3K9ac (RRID: AB_823528, 1:50,000), H3K14ac (RRID: AB_10839410, 1:5,000), H3K18ac (RRID: AB_2783723, 1:30,000), H3K27ac (RRID: AB_10949503, 1:15,000), H3K56ac (RRID: AB_10548193, 1:1,000), histone H4 (RRID: AB_1147658, 1:1,000), H4K5ac (RRID: AB_11217428, 1:100,000), H4 Antibodies against Gli1 (sc-515751, 1:1,000), actin (RRID: AB_2223345, 1:3,000), and tubulin (RRID: AB_1130901, 1:3,000) were purchased from Santa Cruz Biotechnology. Antibodies against HAT1 (RRID: AB_2116435, 1:3,000) were purchased from Proteintech. For *Ptch;p53* SD-CSC tumors shown in Figs. 2B, 4C, and 4F, same ß-ACTIN loading control image (indicated by *) is shown because same samples were loaded on multiple gels on the same day and membranes were cut into multiple pieces to probe different proteins shown.

### IHC

IHC was performed following standard protocols using paraffin sections with the indicated primary antibodies: NEUN (Millipore, RRID: AB_2298772, 1:200).

### Statistical Analyses

Statistical comparisons were performed using GraphPad Prism (RRID:SCR_002798) or R Project for Statistical Computing (RRID:SCR_001905). Values and error bars represent the mean ± SEM. The respective number of replicates (*n*) values are indicated in figures or in figure legends. *P* values were determined by an appropriate statistical test, such as Student *t* tests or ANOVA, with multiple comparison corrections, as indicated in the figure legend. Fisher exact test was used to test the difference in the rate of acquired mutations in the different subtypes.

### Data Availability

Exome-seq and methyl-seq data are available through the SRA portal (https://www.ncbi.nlm.nih.gov/bioproject/PRJNA793810). RNA-seq data are available through the SRA portal (https://www.ncbi.nlm.nih.gov/bioproject/PRJNA834841).

The publicly available human gene expression profiling dataset GSE85217 (https://www.ncbi.nlm.nih.gov/geo/query/acc.cgi?acc=GSE85217) was downloaded from Gene Expression Omnibus and used to analyze BRD2 and BRD4 expression levels in different medulloblastoma subtypes and survival (33). The proteome profiling data have been deposited to the MASSIVE repository (MSV000087151).

## Results

### Acquired Mutations in the SHH Pathway Occur Only if CSCs Depend on the SHH Pathway

To experimentally test our hypothesis that the dependence of CSCs, rather than bulk tumor cells, to the targeted pathway determines the molecular mechanism of therapeutic resistance (Fig. 1A), we first used an autochthonous SHH medulloblastoma mouse model (*FSmoM2;hGFAP-cre*) in which the activated SMO (SMO-M2) allele is conditionally expressed upon Cre recombinase expression (34). We previously reported that 100% of *FSmoM2;hGFAP-cre* mice develop medulloblastomas and hydrocephalus and die around weaning age (18). Furthermore, all medulloblastomas that arise from transformed NSCs in *FSmoM2;hGFAP-cr*e are SI-CSC tumors, due to Cre expression in the neuroepithelium (35). CSCs in *FSmoM2;hGFAP-cre* medulloblastomas depend on bFGF/EGF and not SHH, similar to their cell of origin (NSCs), and they are insensitive to SHH inhibition (18), even though the SHH pathway is highly activated in bulk tumor cells in this model (Supplementary Fig. S1A). When *FSmoM2;hGFAP-cre* pups were treated with LDE225, we observed milder hydrocephalus and consistently smaller brains in the treated mice (Fig. 1B). However, SMOi treatment alone was not sufficient to significantly increase survival in *FSmoM2;hGFAP-cre* mice (Fig. 1C), even though LDE225 treatment had the intended on-target effect of reducing SHH pathway activity *in vivo*, as demonstrated by the lower GLI1 expression level in treated mice (Fig. 1D).

To validate our findings in an independent model, we performed the same experiment in a well-characterized transplantable model of *Ptch;p53* SHH medulloblastoma (18). In this model*, Ptch* and *p53* mutations are germline, and cellular transformation can occur in either NSCs or CGPs in different mice, resulting in either SI-CSC or SD-CSC medulloblastomas, respectively (18). We isolated primary tumorspheres from spontaneous *Ptch;p53* medulloblastomas and characterized each medulloblastoma as either the SD-CSC or SI-CSC subtype by *in vitro* culture phenotypes [Supplementary Table S1 (18)]. In addition, we performed whole-genome bisulfite sequencing analysis to test their epigenomic differences and observed that SD-CSCs clustered together but away from SI-CSCs (Supplementary Fig. S1B; Supplementary Table S2). In parallel, we injected primary, uncultured tumorspheres from three independent *Ptch;p53* MBs into *NOD-SCID;Il2gr^−^^/^^−^ (NSG)* host mice to generate three allograft cohorts with isogenic tumors (Supplementary Fig. S1C). When the tumor volume was approximately 100 mm^3^, we divided each cohort into two groups, treated the mice with either vehicle or LDE225, and measured tumor growth. All mice initially responded to LDE225 treatment, and the tumor volume was reduced 3–4 days after the first treatment (Supplementary Fig. S1D). When the tumor volume had decreased to <50 mm^3^, we halted treatment and evaluated the mice for tumor recurrence. When the tumors rebounded (to >100 mm^3^), we resumed treatment. In all SI-CSC medulloblastomas, resistant tumors grew immediately after the initial debulking phase (Supplementary Fig. S1D). In contrast, in most SD-CSC medulloblastomas, multiple rounds of SMOi treatment were required for the emergence of resistant tumors, suggesting that they required acquisition of additional mutations (Supplementary Fig. S1D). Consistently, mice bearing *Ptch;p53* SD-CSC intracranial tumors tended to survive longer than vehicle-treated mice, but this difference did not reach statistical significance (*P* = 0.1591; Supplementary Fig. S1E).

Next, we analyzed each resistant tumor for treatment-induced mutation through whole-exome sequencing (WES; coverage >120×) from both *Ptch;p53* and *FSmoM2;hGFAP-cre* medulloblastomas. To account for baseline mutational profile differences in each spontaneous *Ptch;p53* tumor, we compared control- and SMOi-treated medulloblastomas from each cohort separately. Compared with control-treated medulloblastomas within each cohort, SMOi-treated tumors exhibited 14 to 146 new SNPs with allele frequency changes >20% (Supplementary Fig. S1F; Supplementary Tables S4 and S5). As anticipated, acquired mutations in SHH pathway genes were detected in SMOi-resistant *Ptch;p53* SD-CSC tumors (four of the six tested, 66.7%; Fig. 1F–H; Supplementary Fig. S1G; Supplementary Table S3). These mutations included a mutation in the *Smo* gene, SmoL416F, which corresponds to the L412F mutation in humans (Fig. 1G); *Gli2* amplification (Fig. 1H); and Mycn amplification (Fig. 1F). These SHH pathway mutations have been previously reported in SMOi-resistant medulloblastomas and BCCs (5, 10, 11). In contrast, none of the *Ptch;p53* SI-CSC SMOi-resistant tumors had mutations in the SHH pathway (zero of three *Ptch;p53* medulloblastomas; Supplementary Table S4). Histologically, control MBs were indistinguishable from LDE225-resistant *Ptch;p53* medulloblastomas of both the SI-CSC and SD-CSC subtypes (Supplementary Fig. S1H).

To validate this finding in an independent model, we performed additional WES from seven *FSmoM2;hGFAP-cre* medulloblastomas. Remarkably, none of the seven LDE225-treated *FSmoM2;hGFAP-cre* medulloblastomas had acquired mutations in the SHH pathway (Fig. 1E; Supplementary Fig. S1I; Supplementary Table S5). Together, drug-induced mutations in the SHH pathway were observed in four of six versus zero of 10 (66.67% vs. 0%, *P* = 0.008) SHH medulloblastomas in the SD-CSC versus SI-CSC subtypes, respectively. These results in two different SHH medulloblastoma models strongly support our hypothesis that treatment-induced mutations in the SHH pathway occur only when the CSCs depend on the SHH pathway.

### SMOi-Treated SI-CSC Medulloblastomas Bypass the SHH Pathway

To confirm that LDE225 treatment suppresses the SHH pathway in both SI-CSC and SD-CSC *Ptch;p53* medulloblastomas, we performed RT-PCR on tumors subjected to both acute and long-term treatment. Acute treatment (3 days *in vivo*) in both subtypes resulted in significant reductions in the levels of SHH pathway genes (Supplementary Fig. S2A), indicating that on-target, therapeutic dosing of LDE225 was achieved in both tumor subtypes. Upon long-term treatment with LDE225, the RNA levels of the known SHH pathway genes *Ptch1, Smo, Gli1,* and *Gli2* were equivalent to those in control-treated tumors in SD-CSC medulloblastomas (Fig. 2A), indicating continued activation of the SHH pathway consistent with acquired mutations in this pathway. One of the SD-CSC tumors with *Gli2* amplification had a higher expression level of *Gli2*. In contrast, in SMOi-resistant *Ptch;p53* SI-CSC tumors*, Gli1* RNA and protein levels were significantly reduced (Fig. 2A and B). These observations strongly suggest that SI-CSC medulloblastomas acquire resistance to SMOis through an alternative mechanism that bypasses the need for SHH pathway activation.

To functionally test this hypothesis, we treated control and SMOi-resistant *Ptch;p53* SI-CSC and SD-CSC tumorspheres with GANT61, a small-molecule inhibitor of GLI1 (36). Others have shown that SMOi-resistant tumors may be inhibited by targeting the downstream effector of the SHH pathway, GLI1 (37). Previously, we reported that *Ptch;p53* SD-CSC tumorspheres but not SI-CSC tumorspheres are sensitive to LDE225 *in vitro* (18). As anticipated, both control and LDE225-resistant *Ptch;p53* SD-CSC tumorspheres were sensitive to GLI1 inhibition, confirming their continued dependence on the SHH pathway (Fig. 2C). In contrast, control and SMOi-resistant *Ptch;p53* SI-CSC tumorspheres were insensitive to GLI1 inhibition (Fig. 2C), confirming their independence from the SHH pathway.

### SI-CSC Medulloblastoma Progenitors Follow an Alternative Differentiation Trajectory Upon SMOi Treatment

To conduct an unbiased investigation of the mechanism by which SI-CSC medulloblastomas continue to grow in the presence of SMOis, we performed RNA-seq analyses of matched control- and LDE225-treated *FSmoM2;hGFAP-cre* SI-CSC medulloblastomas (Supplementary Fig. S2B; Supplementary Table S6). A total of 893 genes were significantly differentially expressed (DE) [FDR < 0.05, log fold change (FC)>1; Supplementary Table S6]. These changes included downregulation of the SHH pathway targets *Gli1, Ccnd1*, and *Myc*; downregulation of the NSC marker genes *Nestin* and *Sox2*; downregulation of the Notch pathway genes *Hes1, Hes5, and Jag1*; downregulation of the cerebellar granule neuronal progenitor marker *Atoh1*; upregulation of cerebellar neuronal differentiation markers *NeuroD1* and *Zic1*; and other alterations (Fig. 2D; Supplementary Fig. S2C and S2D). Collectively, these changes suggested increased neuronal differentiation in LDE225-treated tumors. Consistent with these findings, pathway analyses of DE genes by GSEA, GSVA, and Ingenuity Pathway Analysis showed consistent enrichment of pathways associated with increased neuronal differentiation, reduced proliferation, and reduced Hedgehog signaling (Fig. 2E). IHC analyses comparing vehicle- and LDE225-treated tumors confirmed significantly increased NeuN expression in LDE225-treated tumors (Fig. 2F). Together, these results indicated that SHH pathway activation in *FSmoM2;hGFAP-cre* mice transforms NSCs by maintaining them in a more NSC/early EGL progenitor-like state marked by high *Sox2, Nes,* and *Atoh1* expression and blocking their terminal differentiation into neurons. However, LDE225 treatment enhanced differentiation, resulting in decreased *Sox2, Sox1, Nes,* and *Atoh1* expression and increased *NeuroD1, Zic1,* and NEUN expression (Fig. 2D and F). Note that the expression levels of receptors for NSC growth factors EGF and bFGF were not significantly altered, while *Ptch1* and *Smo* expression was reduced (Fig. 2D). RNA-seq analysis of *Ptch;p53* SI-CSCs showed the same general trend, with significantly reduced *Gli1* expression in SI-CSC tumors but not in SD-CSC tumors (Supplementary Fig. S2E; Supplementary Table S7 and S8).

### Epigenetic Reprogramming in SMOi-Resistant SI-CSC Medulloblastomas

**FIGURE 3:**
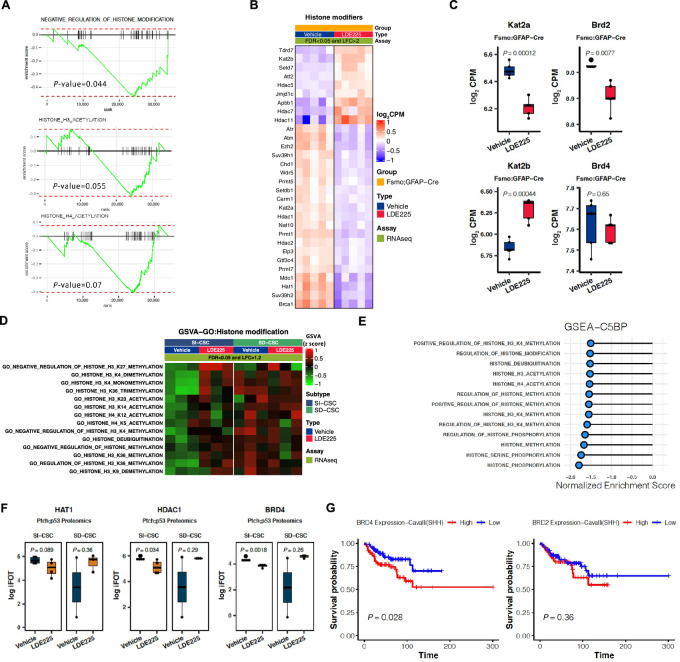
Unbiased RNA-seq and proteomics analyses indicate altered histone modification pathways in SMOi-resistant tumors. **A,** GSEAs showing negative enrichment of histone modification gene sets in LDE225-resistant *fSmoM2;hGFAP-cre* SI-CSC medulloblastomas. **B,** Heatmap showing top DE histone modification-related genes in *fSmoM2;hGFAP-cre* medulloblastomas. FDR < 0.05 and log fold change>2. Expression values were normalized and centered. **C,** Expression levels of *Kat2a, Kat2b, Brd2, and Brd4* in vehicle versus LDE225-treated *fSmoM2;hGFAP-cre* medulloblastomas. Box represents log_2_-scaled CPM range in RNA-seq data. Central line represents the mean. *P* values were calculated using two-tailed Student *t* test. **D,** GSVA analyses (GSVA enrichment score FC>1.2 and FDR<0.05) showing enrichment of histone modification gene sets in LDE225-resistant SI-CSC *Ptch;p53* but not LDE225-resistant SD-CSC *Ptch;p53* tumors. **E,** GSEAs (NES>1.5) of proteomics data showing negative enrichment of histone modification gene sets in LDE225-resistant *Ptch;p53* SI-CSC medulloblastomas. **F,** Expression levels of HAT1, HDAC1, and BRD4 in vehicle versus LDE225-treated *Ptch;p53* SI-CSC and SD-CSC tumors. Box represents log_2_-scaled iFOT range in proteomics data. Central line represents the mean. *P* values were calculated using two-tailed Student *t* test. **G,** Kaplan–Meier survival curves from analyzing a human medulloblastoma dataset [GSE85217, Cavalli et al., ref(33)] for SHH medulloblastoma patients expressing high or low levels of *BRD2* and *BRD4*, using median cutoff. Data represent log_2_ signal. *P* values were calculated using ordinary one-way ANOVA with Tukey multiple comparisons test. SHH subgroup: *n*  =  223.

Other investigators have also reported the emergence of LDE225-resistant *Ptch;p53* medulloblastomas that do not acquire mutations in the SHH pathway (10), but the mechanism underlying these resistant tumors is not well understood. Hence, we next focused on elucidating the mechanism underlying SMOi resistance in the absence of activating mutations in the SHH pathway. We hypothesized that SI-CSC medulloblastomas continue to grow in the presence of LDE225 by bypassing the SHH-dependent CGP-like cell state through epigenetic reprogramming of bulk tumor cells (Fig. 1A). To test this hypothesis, we performed targeted pathway analyses of DE genes identified by RNA-seq in vehicle- versus LDE225-treated *FSmoM2;hGFAP-cre* medulloblastomas. Multiple pathways associated with histone acetylation and methylation were significantly enriched [normalized enrichment score (NES)>2; Fig. 3A; Supplementary Fig. S2F], suggesting significant epigenetic reprogramming in LDE225-treated tumors. Because the role of differential histone methylation during development and drug resistance is well established (38, 39), we focused our analysis on histone acetylation changes. The RNA-seq results from *Fsmo;hGFAP-Cre* medulloblastomas showed significant downregulation of histone acetyltransferases (HAT: *Hat1* and *Kat2a/GCN5*), the histone deacetylase (HDAC) *Hdac1*, and the histone acetylation reader *Brd2* between vehicle- and LDE-treated samples (Fig. 3B and C; Supplementary Fig. S2F–S2H). Interestingly, the *Kat2b/PCAF, HDAC5, 7*, and *11* RNA levels were increased in resistant tumors, and the RNA level of *Brd4*, previously reported to mediate SMOi resistance in SHH MBs by upregulating *Gli1* expression (40), was not significantly altered (Fig. 3B and C). To determine whether the changes in these epigenetic regulator expression patterns are generic responses to LDE225 in all medulloblastomas, we also analyzed *Ptch;p53* SI-CSC and SD-CSC medulloblastomas treated with LDE225 by RNA-seq. While SI-CSC *Ptch;p53* MB samples exhibited differential expression of the genes in histone acetylation and methylation pathways in LDE225-treated tumors, there was no significant difference between vehicle- and LDE-resistant SD-CSC medulloblastomas (Fig. 3D), suggesting that these processes are not generic responses to SMOi treatment in all medulloblastoma subtypes; rather, they are specifically associated with SI-CSC tumors.

To independently test whether similar epigenetic changes are observed at the protein level, we performed a proteomics analysis of *Ptch;p53* SI-CSCs and SD-CSCs treated with LDE225 (Supplementary Fig. S3A–S3C). A total of 7,540 gene protein products (GP) were recovered from five control and four LDE225-treated samples by mass spectrometry–based label-free proteome profiling. We identified 244 GPs that were significantly upregulated and 203 GPs that were significantly downregulated in SMOi-resistant tumors by LDE225 treatment (Supplementary Table S9). Pathway analyses showed significant upregulation of proteins associated with macromolecule degradation and metabolism of amino acids, lipids, and glucose; downregulation of DNA repair pathways/cell-cycle progression; and other alterations (Supplementary Fig. S3D). Consistent with the RNA-seq results, biological process gene sets associated with histone acetylation, methylation, and phosphorylation were enriched with DE proteins in LDE225-resistant SI-CSC samples (Fig. 3E; Supplementary Fig. S3E). In addition, ATOH1, GLI2, and SOX1 protein levels were significantly reduced in SI-CSC medulloblastomas but not in SD-CSC medulloblastomas that were resistant to LDE225 (Supplementary Fig. S3F). The HAT1 protein level was reduced, although not significantly (*P* > 0.05); however, HDAC1 and BRD4 levels were significantly downregulated at the protein level (Fig. 3F), suggesting potential posttranscriptional regulation of the protein expression of some genes.

Because *Brd2* and *Brd4* expression was reduced in LDE225-treated *Ptch;p53* MBs at either the RNA or protein level, we analyzed the expression levels and prognostic value of Brd2/4 in a published gene expression and survival dataset of human medulloblastoma (33). Although the *BRD4* level did not stratify patient survival in the total medulloblastoma cohort (Supplementary Fig. S4A), a lower *BRD4* expression level was associated with significantly better survival in the SHH medulloblastoma subgroup (Fig. 3G). While lower *BRD2* expression predicted better survival in the total medulloblastoma cohort, lower *BRD2* expression was not significantly associated with better survival in the SHH subgroup (Fig. 3G; Supplementary Fig. S4B).

### Altered Histone Code in LDE225-Resistant SI-CSC Medulloblastomas

**FIGURE 4:**
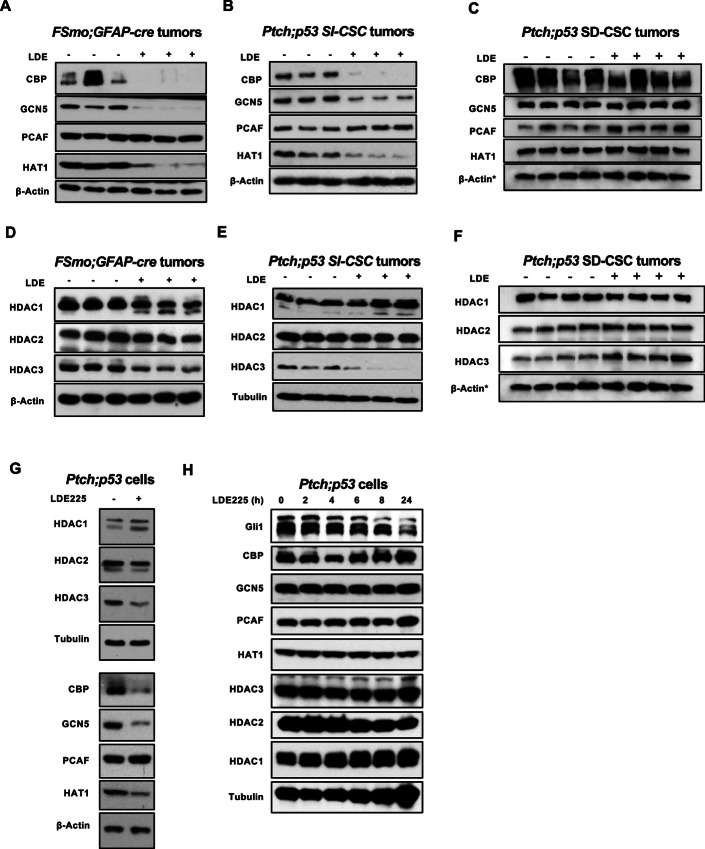
Chronic SMOi treatment reduces specific HAT and HDAC protein levels in SI-CSC medulloblastomas *in vitro* and *in vivo*. Western blot analyses of histone acetyl transferases and histone deacetylases in vehicle versus LDE225 -resistant *fSmoM2;hGFAP-cre* and *Ptch;p53* SHH medulloblastomas. **A** and **D,***fSmoM2;hGFAP-cre SI-CSC***,** (**B** and **E**) *Ptch;p53* SI-CSC medulloblastomas and (**C** and **F**) *Ptch;p53* SD-CSC medulloblastomas. *ß-ACTIN loading control is the same as shown in Figs. 4C and F as the same blot was stripped and probed with multiple antibodies due to limited sample amounts. **G,** Western blot analyses of LDE225-resistant *Ptch;p53* SI-CSC tumorsphere cells treated with LDE225 for long term (>2 weeks) *in vitro* show the same HAT and HDAC expression changes. **H,** Acute (24 hours) treatment of *Ptch;p53* SI-CSC tumorsphere cells show no significant changes in CBP, GCN5, or HAT1 protein levels in response to short-term LDE225 treatment.

Our high-throughput analyses at the RNA and protein levels showed consistent alterations in histone modification pathways in LDE225-resistant SI-CSC medulloblastomas in both the *FSmoM2;hGFAP-cre* and *Ptch;p53* models. To determine the functional consequences of these alterations, we first examined the expression levels of HATs (i.e., writers) and HDACs (i.e., erasers) in an independent cohort of samples by Western blot analysis of both medulloblastoma models. We observed a dramatic reduction in the protein levels of specific HATs (HAT1, CBP, and GCN5 but not PCAF) in SMOi-resistant *FSmoM2;hGFAP-cre* and *Ptch;p53* SI-CSC tumors compared with vehicle-treated tumors. (Fig. 4A and B). This was not a general reduction in all HAT proteins, because the PCAF protein level was unchanged or slightly increased, consistent with the RNA-seq analysis results (Fig. 4A and B). In contrast, there was no significant change in these HAT protein levels in *Ptch;p53* SD-CSC tumors *in vivo* (Fig. 4C). HDAC1 and HDAC2 levels did not differ consistently between the two models, but the HDAC3 protein level was consistently reduced in both SI-CSC medulloblastoma models (Fig. 4D and E). Again, these changes in HDAC levels were not observed in *Ptch;p53* SD-CSC medulloblastomas (Fig. 4F). Notably, these changes in the histone machinery in SI-CSC medulloblastomas were reproducible *in vitro* in long-term LDE225-treated *Ptch;p53* and *FSmoM2;hGFAP-cre* primary medulloblastoma tumorspheres (Fig. 4G), indicating a generalizable epigenetic mechanism that occurs both *in vivo* and *in vitro*. Notably, this response was not an acute response to SMOi treatment, because short-term (up to 24 hours) LDE225 treatment did not reduce the protein levels of HATs or HDACs (Fig. 4H), indicating that these changes are adaptative mechanisms to chronic SMOi exposure and likely contribute to deviations from generating SHH-dependent CGPs in LDE225-resistant SI-CSC medulloblastomas. Together, these observations demonstrated that the downregulation of specific histone acetylation regulators in response to chronic SMOi exposure is a robust mechanism that is readily reproducible in multiple experimental systems *in vivo* and *in vitro*.

### Altered Histone Code and Synthetic Lethality with an HDAC Inhibitor in SI-CSC Medulloblastomas

**FIGURE 5:**
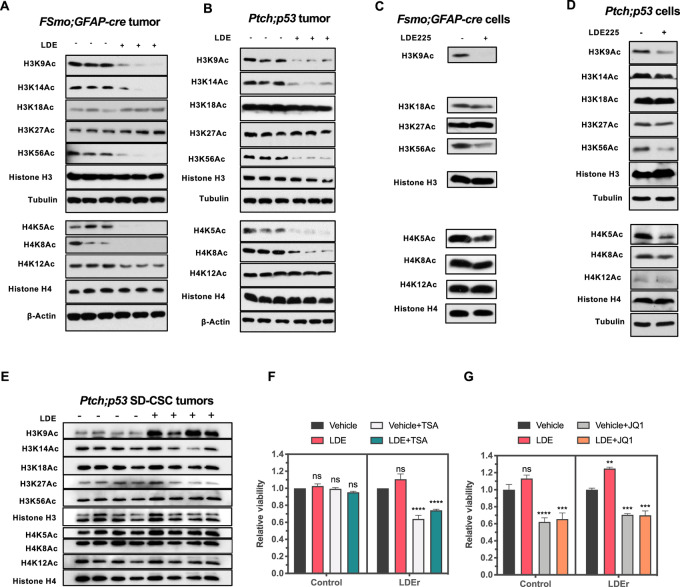
Decreased H3K9, H3K14, H3K56, H4K5, and H4K8 marks in SMOi-resistant SI-CSC medulloblastomas. **A** and **B,** Western blot analyses of different histone acetylation marks comparing vehicle- versus LDE225-treated *fSmoM2;hGFAP-cre* and *Ptch;p53* medulloblastomas *in vivo*. **C** and **D,** Western blot analyses of SMOi-resistant SI-CSC tumorsphere cells from *fSmoM2;hGFAP-cre* and *Ptch;p53* medulloblastoma treated with LDE225 for long term (>2 weeks) *in vitro* show the same HAT and histone acetylation mark changes. **E,** Western blot analyses of different histone acetylation marks comparing vehicle versus LDE225-treated *Ptch;p53* SD-CSC medulloblastoma *in vivo*. **F,** Epigenetically reprogrammed LDE225-resistant SI-CSCs, but not vehicle treated, are sensitive to TSA (10 nmol/L) treatment. **G,** Both control and LDE-resistant SI-CSCs are sensitive to 100 nmol/L JQ1 treatment *in vitro. P* values were calculated using ordinary one-way ANOVA with Sidak multiple comparisons test. *N* = 3; ^****^, *P* < 0.0001; ^*^, *P* < 0.05.

To elucidate the functional outcomes of altered HAT and HDAC protein levels, we analyzed histone marks regulated by HAT1, GCN5, and CBP (41–43). H3K9Ac, H3K14Ac, H3K56Ac, H4K5Ac, and H4K8Ac marks were significantly reduced in both *FSmoM2;hGFAP-cre* and *Ptch;p53* SI-CSC medulloblastoma mouse models treated with LDE225 (Fig. 5A and B). In contrast, H3K27Ac and H4K12Ac levels, which are regulated by other HATs, were not consistently affected (Fig. 5A and B), indicating specific rather than global downregulation of histone acetylation in SMOi-resistant tumors. Furthermore, these histone acetylation changes were reproducible *in vitro* in *FSmoM2;hGFAP-cre* and *Ptch;p53* primary medulloblastoma tumorspheres subjected to long-term treatment with LDE225 (Fig. 5C and D). Importantly, these changes were not observed in *Ptch;p53* SD-CSC tumors (Fig. 5E), which acquire genetically driven resistance to LDE225 through continued activation of the SHH pathway.

Finally, to determine whether the observed dysregulation of the histone acetylation machinery offers an opportunity for synthetic lethality, we treated control and SMOi-resistant isogenic *Ptch;p53* SI-CSC tumorsphere cells with HAT, HDAC, and BET protein inhibitors *in vitro*. Targeting different HAT family members with HATi IV and HATi VIII did not affect the proliferation/survival of either control or SMOi-resistant cells (Supplementary Fig. S5A and S5B). In contrast, treatment with an HDAC inhibitor, TSA (10 nmol/L), significantly reduced the viability of SMOi-resistant SI-CSC cells but did not affect isogenic vehicle-treated cells (Fig. 5F), indicating that reduced histone acetylation is critical to the survival of LDE225-treated SI-CSC medulloblastoma cells *in vitro*.

Considering the clinical interest in BET domain inhibitors for treating medulloblastomas (44, 45), we also tested whether SMOi-resistant *Ptch;p53* SI-CSC medulloblastomas remain sensitive to JQ1, a small-molecule inhibitor of BRD4 and other BET domain proteins. Interestingly, JQ1 significantly reduced the viability of both control and SMOi-resistant SI-CSC tumorspheres (Fig. 5G), suggesting that SMOi-resistant SI-CSC medulloblastomas remain sensitive to BET inhibition. This observation suggests additional mechanisms of action for JQ1 in addition to the regulation of *Gli1* transcription, as reported previously (40).

### Increased Proteasome Activity and Degradation of Specific HATs

**FIGURE 6:**
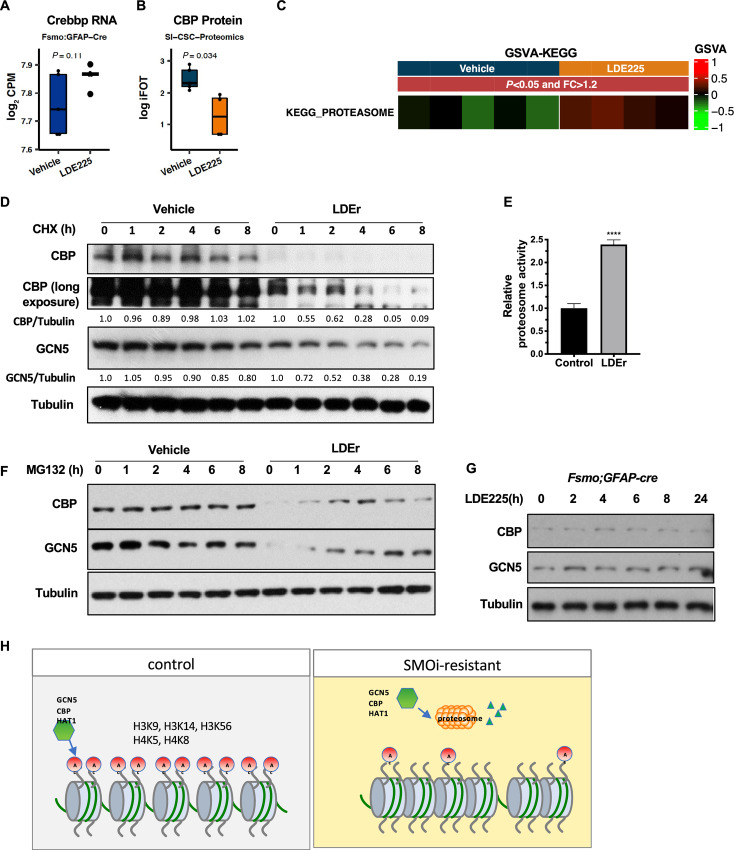
Increased proteasome activity in SMOi-resistant SI-CSC tumors degrade GCN5 and CBP. Histone acetyltransferase *Crebbp*/CBP level is not altered at the RNA level (**A**) but is significantly reduced at the protein level (**B**). Also see Fig. 4 A and B. **C,** Significant enrichment of the Proteasome pathway geneset in proteomics dataset in *Ptch;p53* SMOi-resistant SI-CSC medulloblastoma. **D,** LDE-resistant SI-CSC cells treated with cycloheximide (CHX) show shortened half-life of CBP and GCN5 proteins compared with control. **E,** Proteasome-Glo assay shows increased proteasomal activity in LDE-resistant SI-CSC cells. *N* = 6, ***P < 0.0001, by two-tailed Student *t* test. **F,** Treatment with a proteasome inhibitor, MG132, shows significant recovery of CBP and GCN5 proteirn levels in LDE-resistant SI-CSC cells. **G,** CBP and GCN5 protein levels are unchanged in response to acute (24 hours) LDE treatment in *fSmoM2;hGFAPcre* tumorsphere cells *in vitro*. **H,** A working model of epigenetically reprogrammed therapy resistance in SI-CSCs medulloblastomas.

Because the RNA and protein expression levels of the histone regulatory components Brd2, Brd4, GCN5, and CBP were inconsistent between the RNA-seq and proteomics analysis data (Figs. 3C, 4B, E, 6A, and B; Supplementary Fig. S2H), we hypothesized that a posttranscriptional mechanism may regulate selective histone regulator protein levels. Consistent with this hypothesis, we observed that the Kyoto Encyclopedia of Genes and Genomes “proteasome pathway” was a significantly enriched pathway in LDE225-treated medulloblastomas (Fig. 6C). We first measured the half-life of HAT proteins in control and SMOi-resistant *Ptch;p53* SI-CSC tumorspheres. In control cells, the half-life of CBP and GCN5 was > 8 hours (Fig. 6D). In contrast, the half-life of CBP and GCN5 was approximately 2 hours in SMOi-resistant cells, suggesting that the degradation of CBP and GCN5 was significantly enhanced in LDE225-resistant cells. To determine whether this enhancement was due to the elevated proteasome activity in LDE225-treated cells, we performed a proteasome activity assay and observed that SMOi-treated *Ptch;p53* SI-CSCs had significantly increased proteasome activity compared with control isogenic cells (>2.6-fold, *P* < 0.0001; Fig. 6E). Consistent with this finding, inhibiting the proteasome with MG132 restored GCN5 and CBP protein levels in LDE225-treated SI-CSCs (Fig. 6F). Notably, acute treatment of naïve SI-CSC tumorspheres with LDE225 for 24 hours did not alter the protein levels of CBP and GCN5 (Fig. 6G), indicating that this phenotype is not a direct effect of the drug but rather an adaptive mechanism activated upon chronic exposure to SMOis. Together, these results suggest that *Ptch;p53* SI-CSC medulloblastoma cells epigenetically reprogram bulk tumor cells upon chronic SMOi exposure by elevating proteasome activity to degrade specific HATs, consequently changing the histone code and gene expression in SMOi-resistant SI-CSC tumors (Fig. 6H).

## Discussion

This study advances our understanding of targeted therapy resistance mechanisms in three significant ways. First, we provide evidence that whether a tumor acquires new mutations (a genetic mechanism) in the targeted pathway is dictated by the dependency of its CSCs and not its bulk tumor cells on the selected pathway. Second, we show that it is possible to predict prior to treatment whether a tumor will acquire resistance via a genetic or epigenetic mechanism based on the phenotype of its CSCs. Although the translation of this approach is currently challenging, it has significant clinical implications in terms of guiding second-line therapy selection or designing combination therapies in a timely manner. Third, we reveal a new mechanism of therapeutic resistance: epigenetic reprogramming of bulk tumor cells through changes in histone acetylation via enhanced degradation of specific HATs resulting in an altered histone code. A previous study reported that transformed Smo/Smo medulloblastoma tissues have increased HDAC1, HDAC2, and HDAC3 and decreased HDAC11 expression compared with wild-type or untransformed Smo/Smo cerebellum tissues (46), suggesting that dysregulated histone acetylation may be integral to medulloblastoma formation. Consistently, HDAC inhibitors were identified as potentially efficacious therapies for medulloblastomas in multiple drug screening studies (47, 48). Here, we show that while control-treated SI-CSCs are insensitive to the HDAC inhibitor TSA, LDE225-resistant SI-CSCs are sensitive and suggest combining SMO and HDAC inhibitors for SMOi-resistant SI-CSC tumors. This study paves the way for precision medicine, including the application of targeted therapies and managing therapy resistance, especially in cases where mutations in the targeted pathway are not apparent in the resistant tumors.

The SMO inhibitors vismodegib/GDC-0449 and sonidegib/LDE225 are currently being evaluated for treating SHH medulloblastomas (8, 14–16, 49). A meta-analysis of clinical trials that included medulloblastomas revealed that the combined overall response rate (ORR) for both drugs was 37% for SHH medulloblastomas and 0% for non-SHH medulloblastomas (8). Interestingly, the trial with sonidegib (14 SHH and 60 non-SHH subtypes) was biased toward the adult population (11 adult and 3 pediatric patients) and showed a higher ORR (55%). The patients in the vismodegib trials were more evenly distributed (18 adult and 14 pediatric patients), and the ORR for the SHH subtype of medulloblastoma was 17%. A higher ORR in an adult-biased trial is consistent with our results. We previously showed that the transformation of neuroepithelial cells in the embryonic brain (*FSmoM2;hGFAPCre*) resulted in infantile medulloblastomas that contain SI-CSCs (18), and they are inherently resistant to SMOi, which would lower the ORR. In contrast, the transformation of committed EGL progenitors postnatally (*Fsmo;Atoh1-CreER*) resulted in adult SHH medulloblastomas that contain SD-CSCs (18); these tumors are sensitive to SMOi treatment and require acquired mutations for resistance to emerge, which may explain the higher ORR in the sonidegib trial.

Previously reported mechanisms of resistance to SMOis include mutations or amplification of SHH pathway components (4, 5, 9, 11), activation of the PIK3/AKT (10) or Ras/MAPK (50) pathways, and persistent activation of *Gli1* expression/SHH pathway signaling via upregulated BRD4 activity (40). Here, we demonstrated that acquired mutations in the SHH pathway occur only when CSCs, not bulk tumor cells, depend on SHH signaling. Unlike a previous study implying that elevated BRD4 expression mediates SMOi resistance by activating *Gli1* transcription (40), we report that JQ1 inhibition suppresses SMOi-resistant SHH medulloblastoma in the absence of significant *Gli1* expression. In addition, the majority of targeted therapy resistance mechanism studies in the field rely on bulk tumor analysis. In contrast, this study emphasizes the role of epigenetic cellular heterogeneity and cell state–specific responses to targeted therapy. It provides compelling *in vivo* evidence that in some tumors, CSCs and bulk tumor cells depend on different mitogenic/survival signaling pathways. A significant consequence of this critical difference is that targeted therapies selected by bulk tumor analysis may not be effective against CSCs in some tumors. In these cases, there is no selective pressure for genetic mutations in the CSCs to the targeted pathway (Supplementary Fig. S6). We propose that the ability to predict specific mechanisms of resistance in individual tumors will greatly enhance the personalization of rational combinations or second-line therapies for patients who acquire or show *de novo* resistance to targeted therapy. For example, to address resistance to SMOis in patients with BCC and medulloblastomas (4, 5, 13), second-line therapies inhibiting downstream activators of the SHH pathway, such as GLI, are being investigated (37, 51). However, our study suggests that additional SHH pathway inhibition will be ineffective in SI-CSC–containing tumors (Fig. 2C) and that patients with these tumors may be insensitive to additional SMO or SHH signaling pathway inhibitors such as GANT61 (36, 37). Instead, SI-CSC tumors may be retrospectively identified by decreased histone acetylation as a biomarker (Fig. 5) and treated with HDAC inhibitors or BRD2/4 inhibitors (Fig. 5F and G).

In summary, this study provides a new conceptual framework to understand therapy resistance mechanisms and identifies potential biomarkers to infer whether clinical resistance to SMOis is acquired via genetically or epigenetically driven mechanisms. It will be important to test whether the epigenetic mechanism we discovered (histone acetylation regulation) plays a broader role in therapeutic resistance in other cancer types, particularly in pediatric cancers, where epigenetic mechanisms play critical roles in tumorigenesis (52, 53).

## Supplementary Material

Supplementary Figures and Tables 1-2 Supplementary figures and Tables 1 & 2

Supplementary Table 3 Supplementary Table 3: Mutational profiles for Ptch;p53 SD-CSC tumors

Supplementary Table 4 Supplementary Table 4: mutational profiles for Ptch;p53 SI-CSC tumors

Supplementary Table 5 Supplementary Table 5: mutational profiles for Fsmo;GFAP-cre SD-CSC tumors

Supplementary Table 6 Supplementary Table 6: DEG in LDE vs control treated fSMO;GFAP-cre tumors

Supplementary Table 7 Supplementary Table 7: DEG in LDE vs. control treated SI-CSC Ptch;p53 tumors

Supplementary Table 8 Supplementary Table 8: DEG in LDE vs control in SD-CSC Ptch;p53 tumors

Supplementary Table 9 Supplementary Table 9: Differentially expressed proteins in LDE vs. control treated Ptch;p53 SI-CSC and SD-CSC tumors
