# Supplementary material for: Cancer Stem Cells, not Bulk Tumor Cells, Determine Mechanisms of Resistance to SMO Inhibitors
Source: Cancer Res Commun. 2022 Jun 6;2(6):402–16. doi: 10.1158/2767-9764.CRC-22-0124 (PMC9853917; doi:10.1158/2767-9764.CRC-22-0124)
Supplement: Supplementary Figures and Tables 1-2 — Supplementary figures and Tables 1 & 2 [file crc-22-0124-s01.pdf]

Supplementary Table 1: Characteristics of spontaneous Ptch;p53 MBs used in the study									
**subtype**	**mice ID**	**Ptch;p53**	**gender**	**latency**	**# cells injected**	**Tumor take rate**		**control**	**LDE**
**SI-CSC**	x16650	het:hom	M	60	2X10^6	18/20	90%	6/6	5/5
**SI-CSC**	x17282	het;hom	M	50	3X10^6	18/20	90%	6/6	6/6
**SD-CSC I**	x15924	het:het	F	145	3X10^6	5/10	50%	4/4	1/1
**SD-CSC I**	F3572 (2' of x15924)	het;het			2X10^6	9/20	45%	2/2	0/2
**SD-CSC I**	x15811	het;wt	M	181	3X10^6	5/10	50%	2/4	0/1
**SD-CSC I**	F3590 (2' of x15811)	het;wt			2X10^6	7/20	35%	1/2	0/3
**SD-CSC I**	x15762	het;het	F	218	3X10^6	6/10	60%	3/3	0/3
**SD-CSC I**	x16660	het;wt	F	216	3.8X10^6	14/16	88%	4/4	4/4
**SD-CSC I**	x16598	het;wt	F	242	3.5X10^6	11/16	69%	3/3	3/3
**SD-CSC I**	x16934	het;het	F	193	2X10^6	12/20	60%	1/1	3/3
**SD-CSC II**	F4479	het;hom	M	65	4X10^6	11/20	55%	3/3	3/3
**SD-CSC II**	x16192	het;het	F	221	1X10^6	8/20	40%	3/3	3/3

Supplementary Table 2: Bisulfide sequencing sample information				
**Sample ID**	**Source**	**Tumor Type**	**Cell line**	**Treatment**
LPMeseq1	918 parental	SD-CSC	918	none
LPMeseq2	918 DMSO	SD-CSC	918	DMSO
LPMeseq3	918 LDEr	SD-CSC	918	LDE
LPMeseq4	2869 DMSO	SD-CSC	2869	DMSO
LPMeseq5	2869 LDEr	SD-CSC	2869	LDE
LPMeseq6	17282 parental	SI-CSC	17282	none
LPMeseq7	17282 DMSO	SI-CSC	17282	DMSO
LPMeseq8	17282 LDEr	SI-CSC	17282	LDE
LPMeseq9	9410 DMSO	SI-CSC	9410	DMSO
LPMeseq10	9410 LDEr	SI-CSC	9410	LDE

Supp Fig1

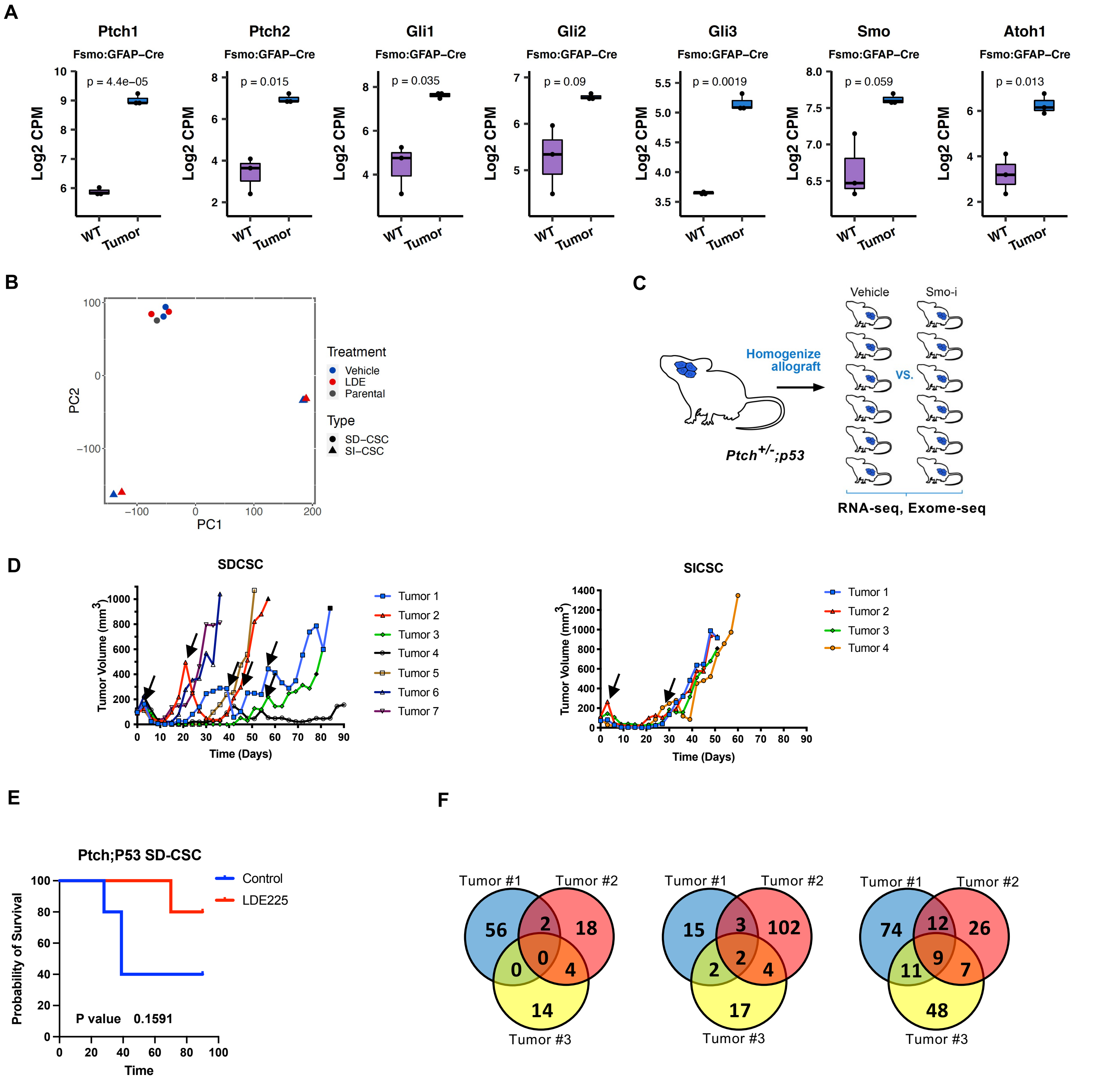
A
Ptch1
Fsmo:GFAP–Cre
p = 4.4e–05
Ptch2
Fsmo:GFAP–Cre
p = 0.015
Gli1
Fsmo:GFAP–Cre
p = 0.035
Gli2
Fsmo:GFAP–Cre
p = 0.09
Gli3
Fsmo:GFAP–Cre
p = 0.0019
Smo
Fsmo:GFAP–Cre
p = 0.059
Atoh1
Fsmo:GFAP–Cre
p = 0.013
Log2 CPM
WT
Tumor
B
Treatment
Vehicle
LDE
Parental
Type
SD–CSC
SI–CSC
PC1
PC2
C
Homogenize allograft
Ptch+/-;p53
Vehicle
Smo-i
VS.
RNA-seq, Exome-seq
D
SDCSC
SICSC
Tumor Volume (mm3)
Time (Days)
Tumor 1
Tumor 2
Tumor 3
Tumor 4
Tumor 5
Tumor 6
Tumor 7
E
Ptch;P53 SD-CSC
Control
LDE225
Probability of Survival
P value 0.1591
Time
F
Tumor #1
Tumor #2
Tumor #3
56
2
18
0
0
4
14
15
3
102
2
2
4
17
74
12
26
11
9
7
48

G

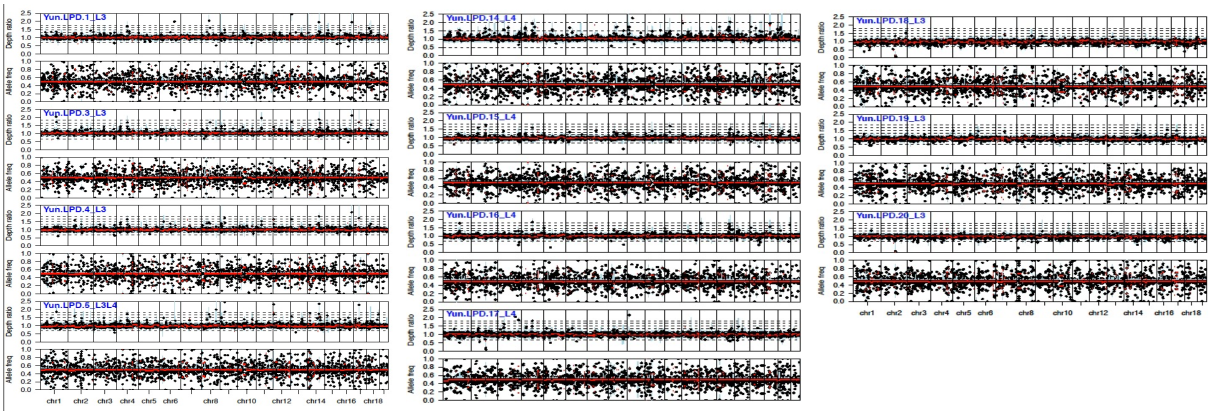

H

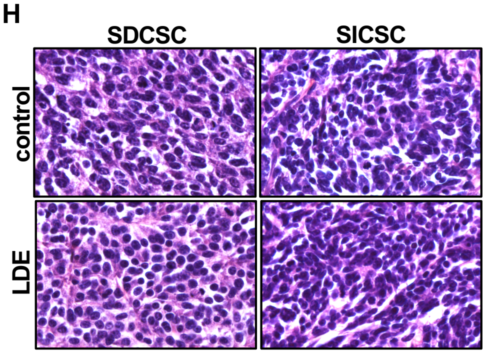

I

Sample	Moderate + High Impact	High Impact
Yun.LPD_LDE.14_L4	10	0
Yun.LPD_LDE.15_L4	26	2
Yun.LPD_LDE.16_L4	7	0
Yun.LPD_LDE.17_L4	12	2
Yun.LPD_LDE.18_L3	6	1
Yun.LPD_LDE.19_L3	7	1
Yun.LPD_LDE.20_L3	8	4
Yun.LPD_Vehicle.1_L3	21	1
Yun.LPD_Vehicle.3_L3	21	2
Yun.LPD_Vehicle.4_L3	10	3
Yun.LPD_Vehicle.5_L3L4	25	3

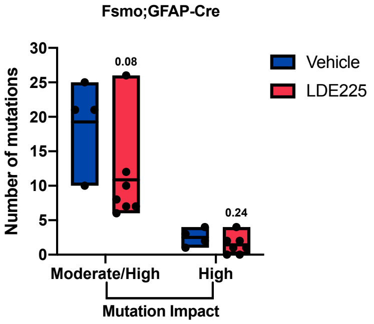

Supplementary Figure 1. ***FsmoM2;hGFAP-cre*** **and** ***Ptch;p53*** **SHH MB models treated with LDE225. A**) Elevated SHH signaling pathway genes in *FsmoM2;hGFAP-Cre MB* compared to wildtype cerebellum at p8. Box represents log_2-scaled CPM range in RNA-seq data. Central line represents the mean. P-values were calculated using two-tailed Student's t-test. **B)** Whole-genome methylation profiles depict significant differences between SI-CSC and SD-CSCs. The dimensionality of methylation profiles of all the samples was reduced using Principal Component Analysis (PCA). A scatter plot on the two-dimensional plane using the first two principal components shows the significant differences between SD-CSCs and SI-CSCs. **C)** A schematic outlining experimental design: spontaneous *Ptch;p53* MBs tumor tissues were isolated, diced into small pieces, and admixed before injecting into a cohort of recipient mice that were randomly divided into two groups (vehicle vs. LDE225(SMOi) treatment). **D)** Representative growth profiles of SD-CSC and SI-CSC tumors treated with LDE225. **E**) Kaplan-Meier survival curve analysis showing survival difference between vehicle and LDE225 treated *Ptch:p53* SD-CSC tumors. **F)** Venn diagrams showing the number of treatment-induced SNPs in each subtype and their overlaps. Numbers indicate SNPs present in LDE-resistant tumors and absent in control-treated tumors from the matching parental tumor. See supplementary tables 3 and 4. **G**) A summary of identified mutations and copy number alterations in the SHH pathway genes in SMOi-resistant *Ptch:p53* SI-CSC and SD-CSC tumors. **H**) H&E staining of control and LDE225 treated tumors at harvest. N=3. **I**) Moderate and high impact mutations identified in vehicle and LDE225 treated *FsmoM2;hGFAP-Cre MB* MB, compared to normal tail DNA by whole exome sequencing analysis. Also see Supp Table 5.

SuppFig2

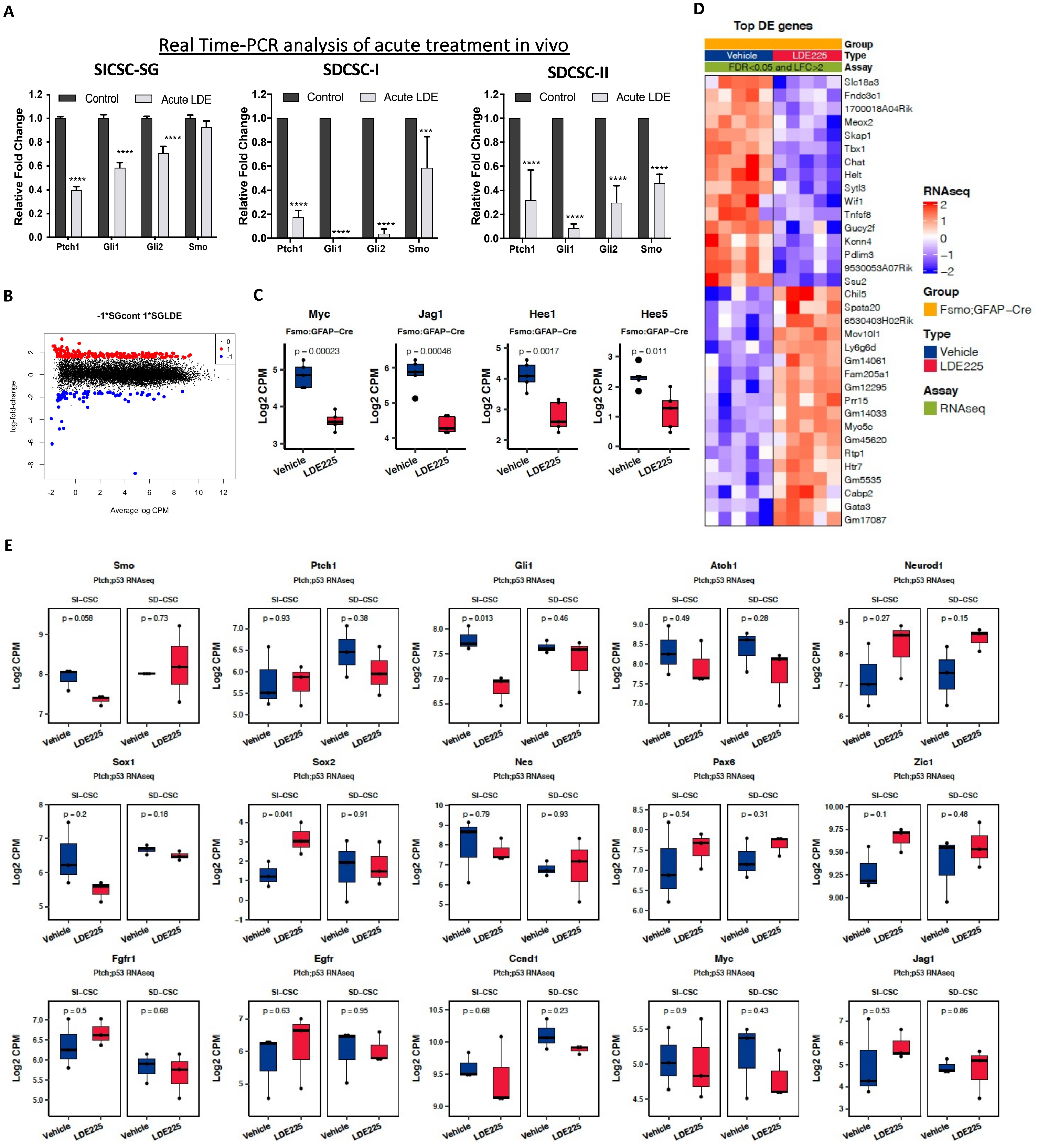
A
Real Time-PCR analysis of acute treatment in vivo
SICSC-SG
SDCSC-I
SDCSC-II
Control
Acute LDE
Relative Fold Change
Ptch1
Gli1
Gli2
Smo
B
-1*SGcont 1*SGLDE
log-fold-change
Average log CPM
C
Myc
Jag1
Hes1
Hes5
Fsmo:GFAP-Cre
p = 0.00023
p = 0.00046
p = 0.0017
p = 0.011
Log2 CPM
Vehicle
LDE225
D
Top DE genes
Group
Type
Assay
Vehicle
LDE225
FDR<0.05 and LFC>2
Slc18a3
Fndc3c1
1700018A04Rik
Meox2
Skap1
Tbx1
Chat
Helt
Sytl3
Wif1
Tnfsf8
Gucy2f
Kcnn4
Pdlim3
9530053A07Rik
Ssu2
Chil5
Spata20
6530403H02Rik
Mov10l1
Ly6g6d
Gm14061
Fam205a1
Gm12295
Prr15
Gm14033
Myo5c
Gm45620
Rtp1
Htr7
Gm5535
Cabp2
Gata3
Gm17087
RNAseq
Fsmo;GFAP−Cre
E
Smo
Ptch1
Gli1
Atoh1
Neurod1
Sox1
Sox2
Nes
Pax6
Zic1
Fgfr1
Egfr
Ccnd1
Myc
Jag1
Ptch;p53 RNAseq
SI−CSC
SD−CSC
Log2 CPM
Vehicle
LDE225

SuppFig2 Cont.

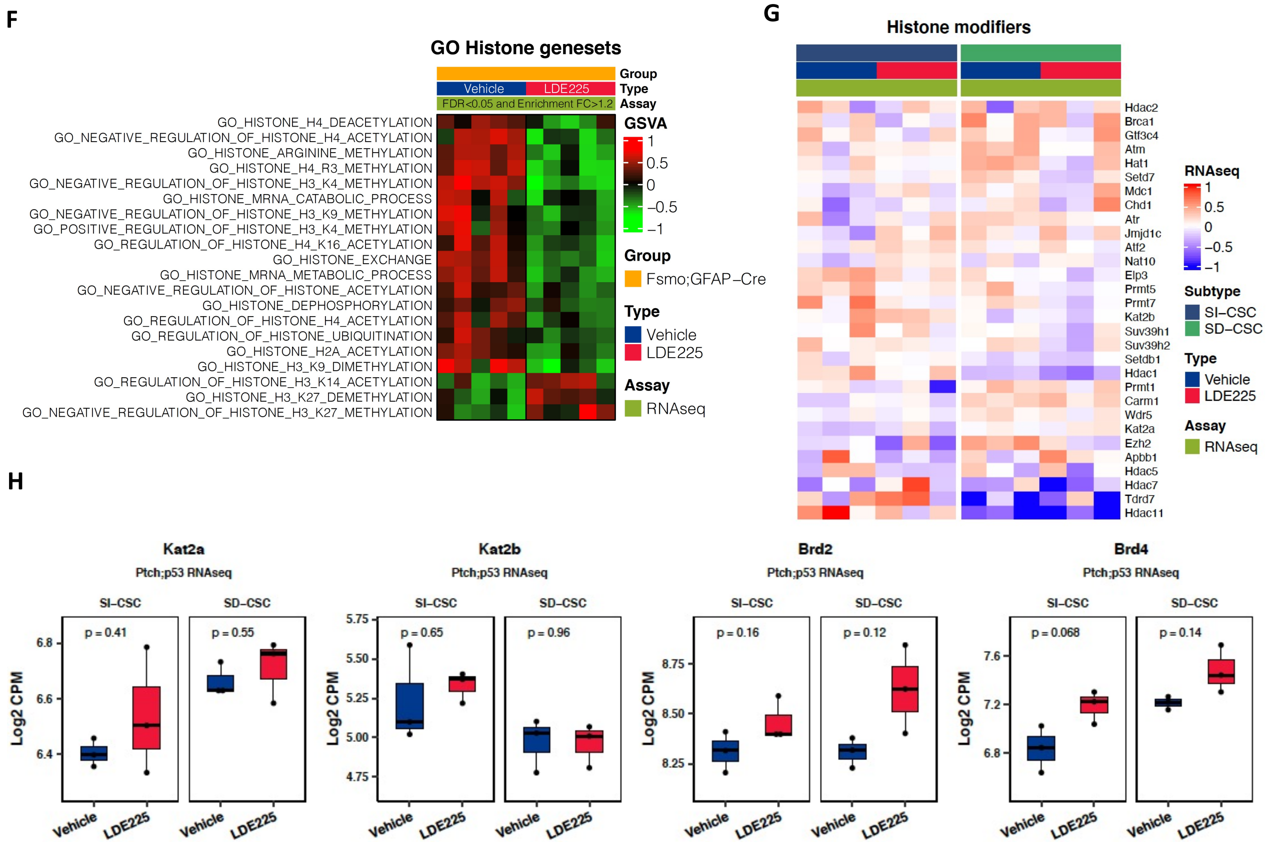

Supplementary Figure 2. Acute and long-term LDE225 treatment reduce SHH , Notch signaling and histone modification gene expression. A) RT-PCR analyses showing on-target response (reduced *Ptch1, Gli1, Gli2, and Smo* RNA levels) in all tumors, regardless of subtype, to acute (2 day) treatment with LDE225. Relative expression levels are normalized to control treated sister allografts. P-values were calculated using ordinary one-way ANOVA with Sidak's multiple comparisons test. N=3, ****P < 0.0001, ***P<0.001. **B**) Mean-difference plot showing RNA-seq expression data of 5 control-treated and 5 LDE225-treated *FSmoM2;hGFAP-Cre MB*. **C**) Expression levels of Myc and Notch pathway genes in vehicle vs. LDE225-treated *FSmoM2;hGFAP-Cre* SI-CSC *MB*s. Box represents log_2-scaled CPM range. Central line represents the mean. P-values were calculated using two-tailed Student's t-test. **D**) Top differentially expressed genes from vehicle vs LDE225 treated *FSmoM2;hGFAP-Cre MB* are represented as Log2-scaled CPM RNA-seq in a heatmap. Each column represents an independent tumor. **E**) Expression levels of *SHH,* neuronal stem cell and differentiation markers in vehicle vs. LDE225-treated *Ptch;P53* MBs. Box represents log_2-scaled CPM range in RNA-seq data. Central line represents the mean. P-values were calculated using two-tailed Student's t-test. . **F**) GSVA analyses (GSVA enrichment score FC>1.2 and p-value <0.05) of vehicle vs. LDE225-treated *FSmoM2;hGFAP-Cre* SI-CSC MBs showing differential enrichment of histone modification gene sets. **G**) heatmap showing histone modification-related genes in *Ptch;P53* SD-CSC MBs. FDR <0.05 and log fold change>2. **H**) Expression levels of *Kat2a, Kat2b, Brd2 and Brd4* in Vehicle vs. LDE225-treated *Ptch;P53* SD-CSC MBs. Box represents log_2-scaled CPM range in RNA-seq data. Central line represents the mean. P-values were calculated using two-tailed Student's t-test.

SuppFig3

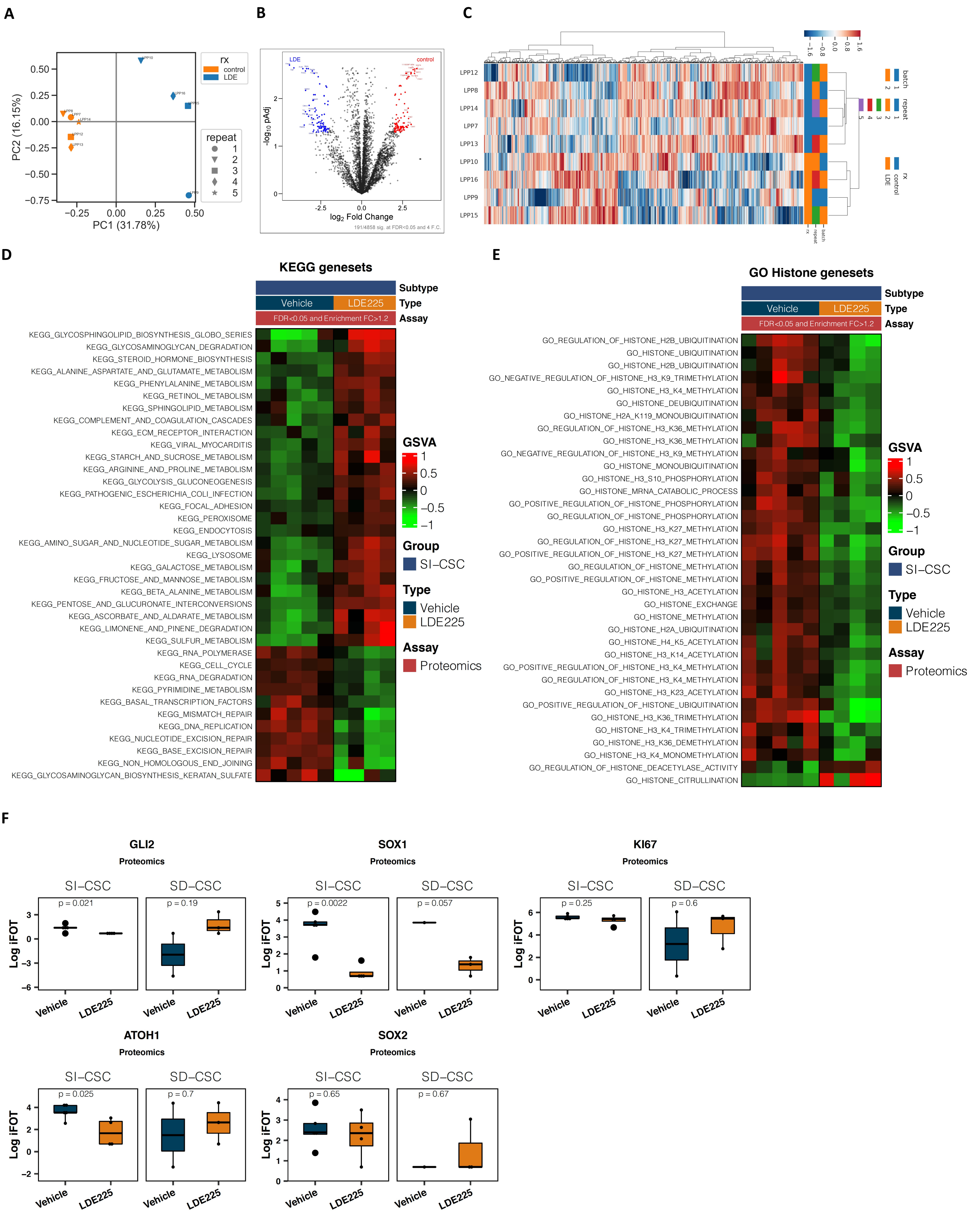
A
PC1 (31.78%)
PC2 (16.15%)
rx
control
LDE
repeat
1
2
3
4
5
B
LDE
control
log2 Fold Change
-log10 pAdj
191/4858 sig. at FDR<0.05 and 4 F.C.
C
LPP12
LPP8
LPP14
LPP7
LPP13
LPP10
LPP16
LPP9
LPP15
batch
repeat
rx
control
LDE
D
KEGG genesets
Subtype
Type
Assay
Vehicle
LDE225
FDR<0.05 and Enrichment FC>1.2
KEGG_GLYCOSPHINGOLIPID_BIOSYNTHESIS_GLOBO_SERIES
KEGG_GLYCOSAMINOGLYCAN_DEGRADATION
KEGG_STEROID_HORMONE_BIOSYNTHESIS
KEGG_ALANINE_ASPARTATE_AND_GLUTAMATE_METABOLISM
KEGG_PHENYLALANINE_METABOLISM
KEGG_RETINOL_METABOLISM
KEGG_SPHINGOLIPID_METABOLISM
KEGG_COMPLEMENT_AND_COAGULATION_CASCADES
KEGG_ECM_RECEPTOR_INTERACTION
KEGG_VIRAL_MYOCARDITIS
KEGG_STARCH_AND_SUCROSE_METABOLISM
KEGG_ARGININE_AND_PROLINE_METABOLISM
KEGG_GLYCOLYSIS_GLUCONEOGENESIS
KEGG_PATHOGENIC_ESCHERICHIA_COLI_INFECTION
KEGG_FOCAL_ADHESION
KEGG_PEROXISOME
KEGG_ENDOCYTOSIS
KEGG_AMINO_SUGAR_AND_NUCLEOTIDE_SUGAR_METABOLISM
KEGG_LYSOSOME
KEGG_GALACTOSE_METABOLISM
KEGG_FRUCTOSE_AND_MANNOSE_METABOLISM
KEGG_BETA_ALANINE_METABOLISM
KEGG_PENTOSE_AND_GLUCURONATE_INTERCONVERSIONS
KEGG_ASCORBATE_AND_ALDARATE_METABOLISM
KEGG_LIMONENE_AND_PINENE_DEGRADATION
KEGG_SULFUR_METABOLISM
KEGG_RNA_POLYMERASE
KEGG_CELL_CYCLE
KEGG_RNA_DEGRADATION
KEGG_PYRIMIDINE_METABOLISM
KEGG_BASAL_TRANSCRIPTION_FACTORS
KEGG_MISMATCH_REPAIR
KEGG_DNA_REPLICATION
KEGG_NUCLEOTIDE_EXCISION_REPAIR
KEGG_BASE_EXCISION_REPAIR
KEGG_NON_HOMOLOGOUS_END_JOINING
KEGG_GLYCOSAMINOGLYCAN_BIOSYNTHESIS_KERATAN_SULFATE
GSVA
Group
SI−CSC
Type
Vehicle
LDE225
Assay
Proteomics
E
GO Histone genesets
GO_REGULATION_OF_HISTONE_H2B_UBIQUITINATION
GO_HISTONE_UBIQUITINATION
GO_HISTONE_H2B_UBIQUITINATION
GO_NEGATIVE_REGULATION_OF_HISTONE_H3_K9_TRIMETHYLATION
GO_HISTONE_H3_K4_METHYLATION
GO_HISTONE_DEUBIQUITINATION
GO_HISTONE_H2A_K119_MONOUBIQUITINATION
GO_REGULATION_OF_HISTONE_H3_K36_METHYLATION
GO_HISTONE_H3_K36_METHYLATION
GO_NEGATIVE_REGULATION_OF_HISTONE_H3_K9_METHYLATION
GO_HISTONE_MONOUBIQUITINATION
GO_HISTONE_H3_S10_PHOSPHORYLATION
GO_HISTONE_MRNA_CATABOLIC_PROCESS
GO_POSITIVE_REGULATION_OF_HISTONE_PHOSPHORYLATION
GO_REGULATION_OF_HISTONE_PHOSPHORYLATION
GO_HISTONE_H3_K27_METHYLATION
GO_REGULATION_OF_HISTONE_H3_K27_METHYLATION
GO_POSITIVE_REGULATION_OF_HISTONE_H3_K27_METHYLATION
GO_REGULATION_OF_HISTONE_METHYLATION
GO_POSITIVE_REGULATION_OF_HISTONE_METHYLATION
GO_HISTONE_H3_ACETYLATION
GO_HISTONE_EXCHANGE
GO_HISTONE_METHYLATION
GO_HISTONE_H2A_UBIQUITINATION
GO_HISTONE_H4_K5_ACETYLATION
GO_HISTONE_H3_K14_ACETYLATION
GO_POSITIVE_REGULATION_OF_HISTONE_H3_K4_METHYLATION
GO_REGULATION_OF_HISTONE_H3_K4_METHYLATION
GO_HISTONE_H3_K23_ACETYLATION
GO_POSITIVE_REGULATION_OF_HISTONE_UBIQUITINATION
GO_HISTONE_H3_K36_TRIMETHYLATION
GO_HISTONE_H3_K4_TRIMETHYLATION
GO_HISTONE_H3_K36_DEMETHYLATION
GO_HISTONE_H3_K4_MONOMETHYLATION
GO_REGULATION_OF_HISTONE_DEACETYLASE_ACTIVITY
GO_HISTONE_CITRULLINATION
F
GLI2
SOX1
KI67
ATOH1
SOX2
Proteomics
SI−CSC
SD−CSC
Log iFOT
Vehicle
LDE225
p = 0.021
p = 0.19
p = 0.0022
p = 0.057
p = 0.25
p = 0.6
p = 0.025
p = 0.7
p = 0.65
p = 0.67

Supplementary Figure 3. Proteomics analysis identifies differentially expressed proteins in LDE225 treated tumors. A) Principal component analysis of the 5 vehicle and 4 LDE225-treated *Ptch;p53* SI-CSC MB samples analyzed by mass spectrometry. **B**) Volcano plots of protein expression changes between vehicle vs LDE225-treated *Ptch;p53* SI-CSC MBs. Blue dots represent the down-regulated proteins and red dots represent upregulated proteins. **C**) Heatmap showing differential expression of proteins in vehicle vs LDE225 treated *Ptch;p53* SI-CSC MBs. **D)** GSVA analyses (GSVA enrichment score FC>1.2 and FDR <0.05) of vehicle vs. LDE225-treated *Ptch;p53* SI-CSC MBs showing differential enrichment of KEGG gene sets. **E)** GSVA analyses (GSVA enrichment score FC>1.2 and FDR <0.05) of vehicle vs. LDE225-treated *Ptch;p53* SI-CSC MBs showing differential enrichment of GO Histone gene sets. **F**) Expression levels of GLI2, SOX1, SOX2, ATOH1 and KI67 proteins in vehicle vs. LDE225-treated *Ptch;p53* SI-CSC and SD-CSC MBs. Box represents $\log_2$-scaled iFOT range. Central line represents the mean. P-values were calculated using two-tailed Student's t-test.

SuppFig4

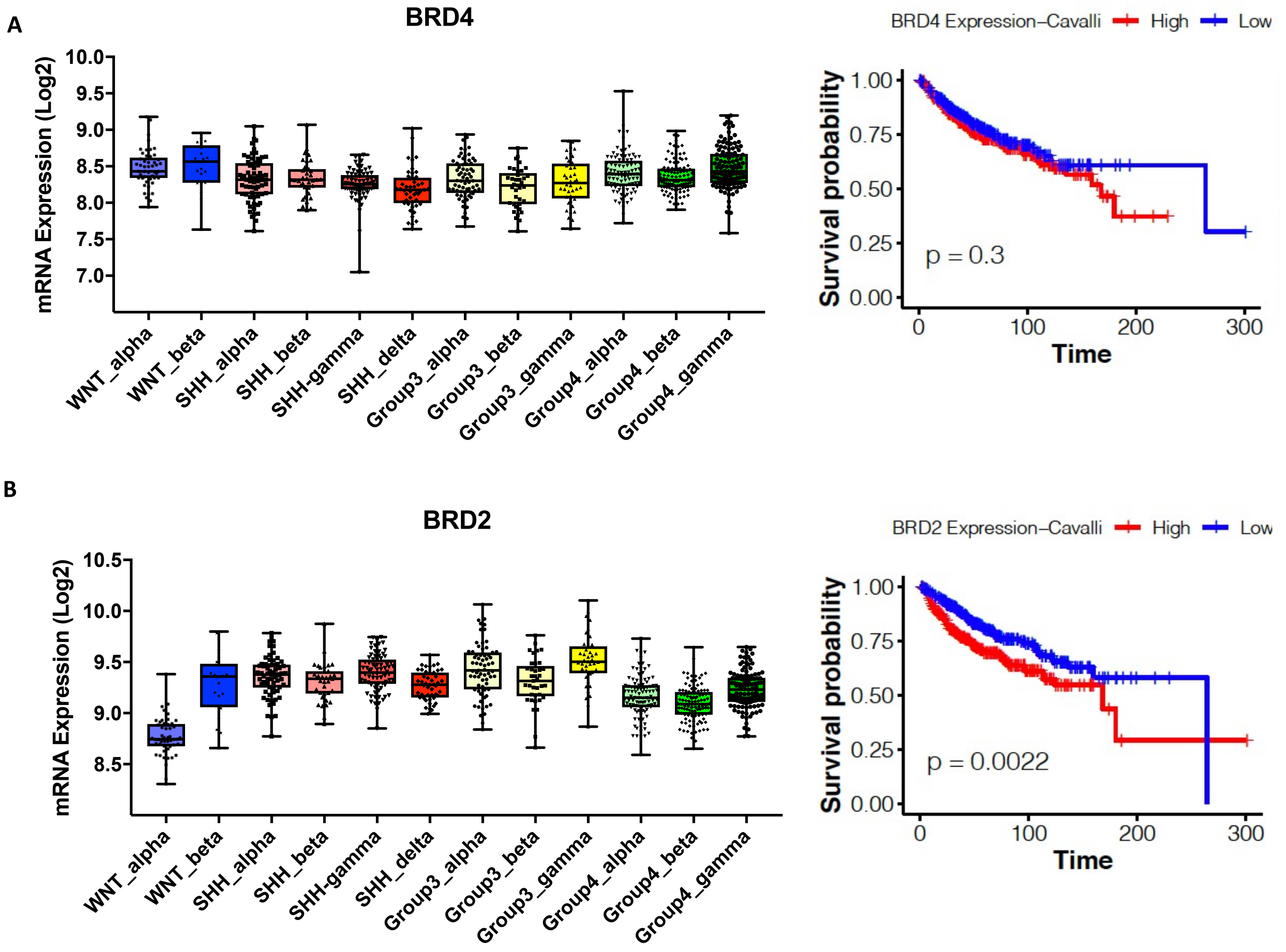

Supplementary Figure 4. Meta-analysis of GSE85217 (Cavalli *et al*) data set using GlioVis. A, B) *BRD4* and *BRD2* mRNA expression levels in different human MB molecular subtypes (left), Kaplan-Meier survival curve analyses of human MB samples by subtypes (right). High and low expression cutoff was set at the median. P-values were calculated using ordinary one way ANOVA with Tukey's multiple comparisons test. (WNT: n = 70; SHH: n = 223; Gp3: n = 144; Gp4: n = 326).

SuppFig5

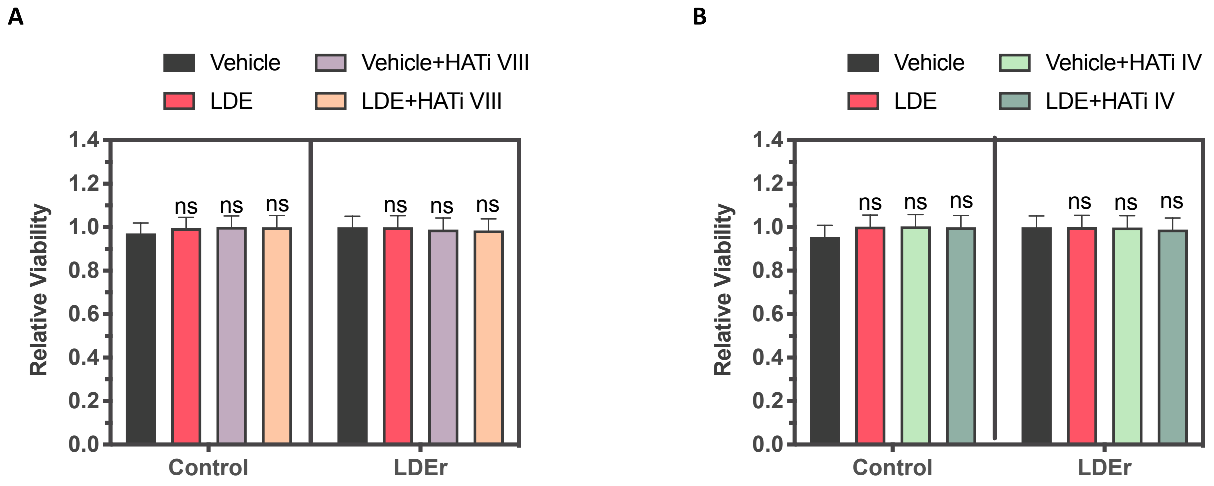

Supplementary Figure 5. HAT inhibitors are ineffective in suppressing LDE-resistant SI-CSC tumorsphere growth in vitro. A, B) Vehicle and LDE-resistant *Ptch;p53* SI-CSC tumorspheres were treated with different HAT inhibitors *in vitro*. N=3.

Supp Fig 6

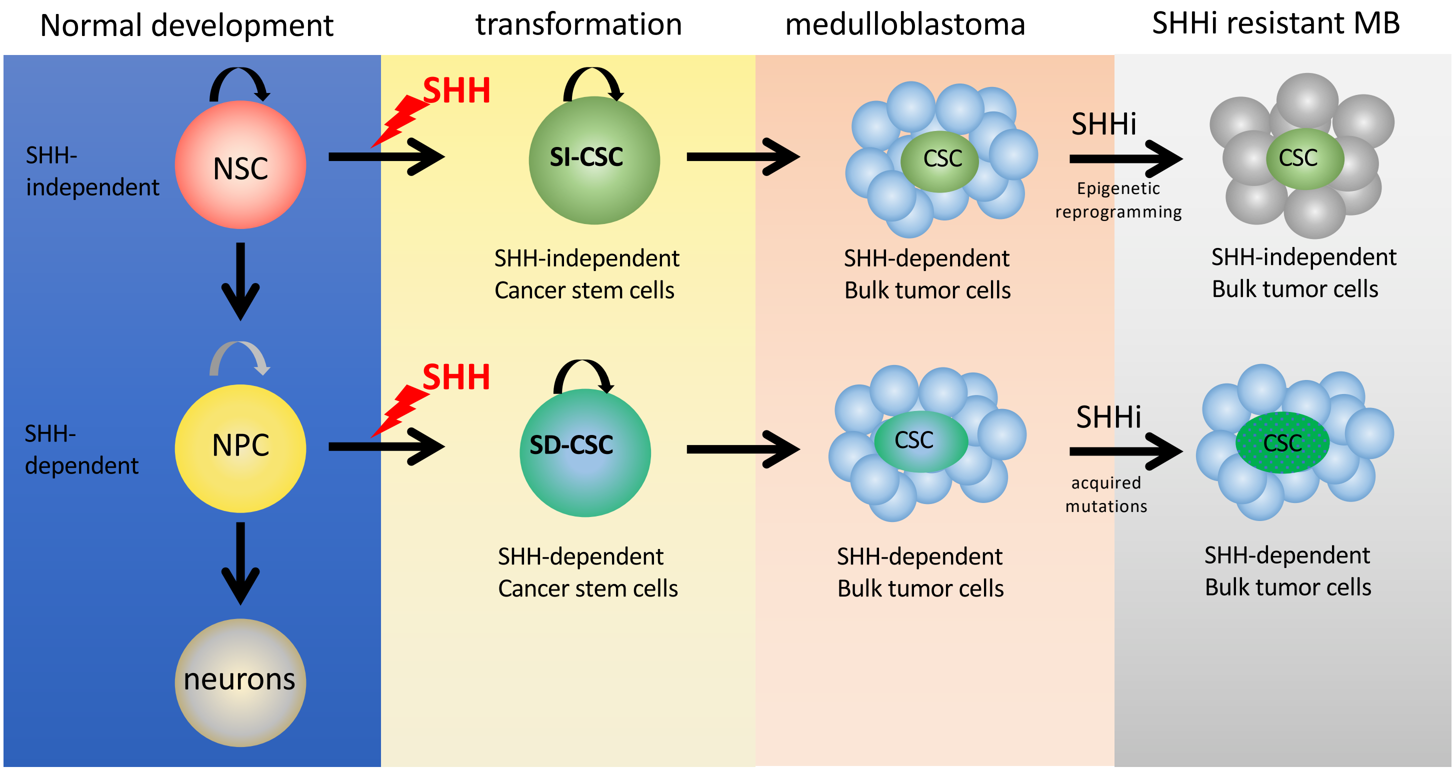

Supplementary Figure 6. A schematic summary of our working model. SHH MB can arise from transformation of either neural stem cells (NSCs, SHH-independent) or EGL neural progenitors (NPCs, SHH-dependent) in vivo. While bulk tumor cells in both tumor type depend on the SHH pathway, only the NPC-derived cancer stem cells (SD-CSC) depend on SHH signaling. SMOi treatment debulks both tumor types; however, acquired mutations within the SHH pathway occurs only in SD-CSC tumors. In SI-CSC subtype, CSCs are insensitive to SMOi and generate bulk tumor cells that deviates from normal NSC maturation through epigenetic reprogramming and generate SHH-independent progenitors/bulk tumor cells upon chronic SMOi exposure.
